# Supplementary material for: Navigating digital frontiers in UAE healthcare: A qualitative exploration of healthcare professionals’ and patients’ experiences with AI and telemedicine
Source: PLOS Digit Health. 2025 Apr 8;4(4):e0000586. doi: 10.1371/journal.pdig.0000586 (PMC11991283; doi:10.1371/journal.pdig.0000586)
Supplement: S1 File — (DOCX) [file pdig.0000586.s001.docx]

**INTERVIEW TRANSCRIPTS**

### ****Participant P.1 Interview Transcript****

**Interviewer:** Thank you for joining us today. To start, can you describe your experience with telemedicine and AI in healthcare?

**P.1:** Sure. Telemedicine has greatly saved my time and finances. It has protected me from the hectic process of traveling to crowded healthcare facilities to see a doctor, where I could even contract other infections. Instead, I can now manage my condition from the comfort of my home.

**Interviewer:** That sounds very convenient. Can you share a specific example where telemedicine has made a significant difference for you?

**P.1:** Definitely. The most significant benefit I associate with telemedicine is how it makes healthcare seamless. For instance, my doctor can monitor my condition remotely without requiring me to visit in person. This was especially crucial during the COVID-19 pandemic when physical contact was restricted to control the spread of the virus. I felt safer knowing that I could still receive care without risking exposure to illness in crowded healthcare facilities.

**Interviewer:** That’s a great example. Have you noticed any other benefits of using telemedicine?

**P.1:** Oh, absolutely. It’s not just about avoiding travel or crowds—it’s also about how much more efficient everything feels. For example, videoconferencing allows me to have meaningful interactions with my doctor without scheduling an in-person appointment. I’ve been able to ask questions, get recommendations, and even adjust my medications, all through these technologies.

**Interviewer:** That’s very insightful. Let’s talk about any concerns you’ve noticed. Are there any challenges you feel come with these technologies?

**P.1:** Yes, there are challenges, and they mostly revolve around data protection. These technologies will only be widely accepted if they address fears associated with protecting users’ data. People need to feel that their digitally collected information is secure and will only be used for treatment or care provision. Many patients are skeptical because they believe that sufficient mechanisms haven’t been put in place to safeguard against data breaches or misuse.

**Interviewer:** That’s an important concern. From your perspective, what do you think could be done to address these data security challenges?

**P.1:** I think it comes down to transparency and accountability. If mechanisms were developed to guarantee data security and transparency, it would address much of the skepticism. People need to trust that their data is stored securely and only accessed when absolutely necessary. Policies need to be clear, and patients should be informed about how their data is being handled. With this kind of assurance, people would trust these technologies more, and their adoption would grow significantly.

**Interviewer:** That’s an excellent point. Do you feel that the benefits of telemedicine and AI outweigh these concerns?

**P.1:** Yes, I believe they do. Despite the concerns, these technologies have already proven how effective they can be in improving healthcare access and convenience. For someone like me, who manages a chronic condition, it’s reassuring to know that I don’t have to physically visit my doctor every time there’s an issue. I also appreciate the time and energy it saves me, allowing me to focus on other parts of my life.

**Interviewer:** It sounds like telemedicine has really improved your healthcare experience. Before we wrap up, is there anything else you’d like to add about your experiences?

**P.1:** Just that these technologies have been a game-changer for me. They’ve made accessing healthcare much easier, and I hope more people will be able to benefit from them as they improve. I’d love to see continued efforts to address challenges like data protection because I believe that with the right measures in place, even the skeptics would embrace these innovations.

**Interviewer:** Thank you so much for your time and for sharing your insights. Your experiences provide a valuable perspective on how these technologies are impacting patients.

**P.1:** Thank you for having me. I hope my input helps to improve how telemedicine and AI are integrated into healthcare.

### ****Participant P.2 Interview Transcript****

**Interviewer:** Thank you for agreeing to participate in this interview. Let’s start with your experience. How would you describe your interactions with AI and telemedicine in your practice?

**Participant P.2:** Honestly, I’ve found telemedicine particularly transformative. It’s allowed us to interact with patients more frequently, improving the doctor-patient relationship. Incorporating telemedicine into our facility has helped us better understand patients’ needs, which ultimately increases the likelihood of achieving desired outcomes.

**Interviewer:** That’s interesting to hear. From your perspective, what specific benefits have you noticed with telemedicine and AI?

**Participant P.2:** One of the standout advantages of AI is its ability to track data across multiple regions and identify patterns that might signal an outbreak. It’s amazing how it can provide crucial insights, enabling faster isolation of cases and preventing the spread of infectious diseases. For example, during the pandemic, tools like these would’ve been invaluable for real-time monitoring and intervention.

**Interviewer:** That’s a great example. What about challenges? Have you noticed any resistance or issues regarding these technologies?

**Participant P.2:** Definitely. Among the many reasons people resist these changes is a lack of awareness about the benefits. For instance, some patients and even clinicians hesitate to embrace AI and telemedicine simply because they don’t fully understand their usefulness. Increased awareness is key to overcoming this hurdle. When stakeholders truly understand how these technologies can improve care, acceptance will naturally follow.

**Interviewer:** So, you believe awareness campaigns are essential?

**Participant P.2:** Absolutely. Awareness is critical, but it must go hand in hand with training. Everyone involved—patients, clinicians, and administrators—needs to see how these tools will improve their experiences and outcomes. Otherwise, the adoption will be slow.

**Interviewer:** Speaking of improvements, in your opinion, how can AI and telemedicine be enhanced to provide even better care?

**Participant P.2:** One way is by making the technology more user-friendly. Many clinicians feel overwhelmed by overly complex systems, which can discourage them from using AI or telemedicine tools. Developers need to involve clinicians in the design process to ensure the systems address real-world needs without adding unnecessary complexity.

**Interviewer:** That’s a valid point. Do you see AI and telemedicine becoming more integrated into healthcare systems in the UAE?

**Participant P.2:** Without a doubt. But for that to happen, we must address not just technical challenges but also cultural ones. Patients and providers alike need to feel confident that these technologies are safe and beneficial. Proper engagement, training, and awareness will go a long way in driving their integration.

**Interviewer:** Thank you for your insights. Before we wrap up, is there anything else you’d like to share about your experience with these technologies?

**Participant P.2:** I’d just emphasize the potential of AI and telemedicine to revolutionize healthcare. But to fully realize this potential, we must address barriers like skepticism and lack of training. If we do that, these tools could truly transform patient care in ways we’ve only imagined before.

**Interviewer:** Thank you so much for your time and valuable input.

**Participant P.2:** You’re welcome. It’s been a pleasure.

**Interview Transcript: Participant P.3**

**Interviewer:** Thank you for taking the time to speak with us today. Could you start by sharing your experiences with telemedicine and AI in healthcare?

**Participant P.3:** Certainly. Consulting my doctor through a video call saves me the struggles of traveling to the healthcare facility, dealing with high traffic on the roads, and queuing for many hours, which would leave me completely exhausted by the end of the day. Telemedicine saves time and money and reduces the risks of infections, especially during flu season. It’s been an incredible convenience, especially when it comes to follow-ups or managing chronic conditions.

**Interviewer:** That’s insightful. How do you feel these technologies have impacted your interactions with healthcare providers?

**Participant P.3:** Engaging in a discussion with a physician who knows you at a personal level makes the patient feel good because it demonstrates that somebody knows you and cares about you and your condition and is determined to ensure you live a quality life. The personalization of communication between the patient and doctor creates a stronger bond, a sense of satisfaction, trust, and confidence in the doctor. Telemedicine has helped maintain this connection, even when physical visits aren’t possible.

**Interviewer:** That’s a great perspective. However, are there aspects of AI and telemedicine that you find less appealing or concerning?

**Participant P.3:** Honestly, I don’t like the idea of programmed machines making critical decisions about my health. My body is so complicated to be diagnosed or even treated by a machine that has been programmed by a person who does not know me or has interacted with me to understand my health and other unique characteristics. I want a human being with whom I can converse and get to know myself in person. Human touch is irreplaceable by AI. While AI can be useful in analyzing data or predicting outcomes, it should not replace the doctor’s role in understanding the patient holistically.

**Interviewer:** That’s a valid concern. What do you think could improve your experience with these technologies?

**Participant P.3:** Transparency is key. Patients need to understand how their data is being used and how secure it is. Building trust in these systems is crucial. Additionally, there should be more patient-centric designs in telemedicine platforms to make them easier to use. If these technologies are designed with both functionality and patient preferences in mind, they could make an even greater impact.

**Interviewer:** Thank you for sharing such detailed insights. Do you have any final thoughts on AI and telemedicine in healthcare?

**Participant P.3:** Yes, I believe these technologies have immense potential, but their success lies in balancing innovation with humanity. They should be tools that enhance the healthcare experience, not replace the personal touch that is so important in medicine. As we move forward, it’s essential to keep the patient at the center of these advancements.

**Interviewer:** Thank you again for your time and thoughtful responses. Your input is invaluable to this research.

**Participant P.3:** I’m happy to contribute. I hope my feedback helps in improving these systems for everyone.

### Interview Transcript: Participant P.4

**Interviewer:** Thank you for joining us today. To start, could you share your overall thoughts on AI and telemedicine in healthcare?

**Participant P.4:** Of course. These technologies bring a lot of potential to the table. Patients also need to understand how these technologies work and how they can benefit from them. They will definitely embrace innovations that enhance efficiency and convenience. If people see tangible improvements in their healthcare experiences, they’re more likely to adopt these innovations.

**Interviewer:** That’s a good point. Can you elaborate on how these technologies could improve efficiency in healthcare settings?

**Participant P.4:** AI can help in the management of appointments and prioritization of cases. This enhances the efficiency of healthcare institutions, reducing delays in appointments and provision of services. For example, using AI to organize appointments based on urgency ensures that patients who need immediate attention are prioritized while also streamlining processes for everyone.

**Interviewer:** It’s clear you see a lot of potential in these tools. How do you think telemedicine, in particular, impacts the patient-doctor relationship?

**Participant P.4:** Telemedicine fosters a connection between patients and doctors, tranquility, and sense of worth. Even though it’s virtual, it allows patients to feel that their needs are understood and that their health is being closely monitored. This connection is invaluable in ensuring patients remain engaged in their care and trust their healthcare providers.

**Interviewer:** Are there any challenges you think need to be addressed to enhance the adoption and effective use of these technologies?

**Participant P.4:** The main challenge is awareness. Patients need to fully understand what these technologies offer and how they can improve their care. Once this understanding is there, it becomes easier to overcome resistance to change. Additionally, healthcare professionals need proper training to maximize the potential of AI and telemedicine. These tools are only as effective as the people using them.

**Interviewer:** That’s insightful. Finally, what do you think would help patients feel more confident in using AI and telemedicine?

**Participant P.4:** Transparency and education are key. When patients understand the benefits and are confident that their data is safe, they’re more likely to trust these systems. It’s also crucial for healthcare providers to engage with patients actively, helping them see the human side of these innovations.

**Interviewer:** Thank you for your thoughtful responses. Your insights will be instrumental in shaping how we understand the role of AI and telemedicine in healthcare.

**Participant P.4:** I’m glad I could contribute. I hope this research helps in creating more patient-centered solutions for everyone.

### Interview Transcript: Participant P.5

**Interviewer:** Thank you for participating in this interview. To begin, could you tell me how telemedicine has impacted your daily life?

**Participant P.5:** My ability to consult my doctor right from here has been a game changer for me, thanks to telemedicine. I rarely travel for consultations, which has greatly helped me remain more productive and efficiently plan my daily routines. It has eliminated the need to take time off work or arrange transportation for minor consultations, which used to be a hassle.

**Interviewer:** That sounds convenient. Could you elaborate on how telemedicine has influenced your healthcare experience overall?

**Participant P.5:** It has significantly enhanced the way I manage my health. I receive regular updates about my health, including when to take or change the medication, what dietary and lifestyle choices I should embrace, all aiming to enhance my health outcomes. In case I notice peculiar symptoms or reactions to food, environment, or drugs, I inform my doctor, who calls me and gives appropriate recommendations. This digital health technology gives a sense of peace. Knowing that my health is being monitored closely without the need for frequent in-person visits has been incredibly reassuring.

**Interviewer:** That’s fascinating. How would you describe the communication with your doctor through telemedicine?

**Participant P.5:** It’s seamless. My doctor is just a call or message away. This kind of connection ensures that I stay on track with my health goals and that any potential issues are addressed immediately. For example, when I noticed a reaction to a particular medication, I informed my doctor through the platform, and they quickly adjusted the prescription. It’s like having a safety net for my health.

**Interviewer:** What do you think can be improved to make your telemedicine experience even better?

**Participant P.5:** I think the system works well, but there’s always room for improvement. For instance, more awareness about how to use these platforms would help others who might be hesitant or unsure. Also, ensuring data security and privacy is essential. If people feel safe using telemedicine, they’ll embrace it wholeheartedly.

**Interviewer:** Thank you for sharing your experiences. Your insights will help us understand the patient perspective on telemedicine.

**Participant P.5:** I’m glad to help. I hope this research contributes to making telemedicine even more effective and accessible.

### Interview Transcript: Participant P.6

**Interviewer:** Thank you for joining us today. To start, could you share your overall experience with digital health technologies like telemedicine?

**Participant P.6:** Certainly. Digital health technologies such as telemedicine have now made it easier for us to transfer some of the responsibilities to the patients. This way, the patients feel they are participating in efforts to improve their well-being, thus boosting their understanding of why specific decisions have been made. It has brought a sense of shared responsibility to the healthcare process, which I think is crucial for better outcomes.

**Interviewer:** That’s an interesting point. Could you elaborate on how these technologies have changed your interactions with patients?

**Participant P.6:** Absolutely. Telemedicine has created a more interactive relationship between us and the patients. For instance, when I provide them with resources or advice through the platform, I can see that they are more engaged and proactive in managing their health. It’s no longer a one-way communication where the doctor simply tells them what to do. Instead, we discuss their health as partners, and they often provide valuable input that helps in decision-making.

**Interviewer:** Have you noticed any challenges that patients face when engaging with these technologies?

**Participant P.6:** Yes, there are challenges. Some patients find it difficult to navigate the technology at first, especially older individuals or those who are less tech-savvy. But once they understand how it works, most of them appreciate the convenience and control it gives them. The key is providing clear instructions and being available to support them during the transition phase.

**Interviewer:** What do you think could be improved to enhance the effectiveness of these technologies?

**Participant P.6:** I believe more awareness and training for both patients and healthcare professionals could make a big difference. Patients need to see how these tools benefit them, and professionals need to fully integrate them into their practices. Additionally, addressing privacy concerns and ensuring secure data handling are critical. Patients need to trust that their information is safe and that the system works in their favor.

**Interviewer:** That’s very insightful. Do you think digital health technologies are here to stay, and if so, what do you envision for their future?

**Participant P.6:** Without a doubt, these technologies are here to stay. They are the future of healthcare, especially as the world becomes more interconnected and reliant on technology. I think we’ll see even more advanced features in telemedicine and AI, which will further empower both patients and healthcare providers. The goal should always be to enhance patient outcomes while maintaining the human touch in care delivery.

**Interviewer:** Thank you so much for your time and thoughtful responses. Your insights are invaluable for our study.

**Participant P.6:** I’m happy to contribute. I hope this research leads to better adoption and implementation of these technologies.

### Interview Transcript: Participant P.7

**Interviewer:** Thank you for participating in our study. Could you begin by sharing your thoughts on how AI and telemedicine are impacting healthcare delivery?

**Participant P.7:** Sure. AI is capable of analyzing a patient’s historical data and alerting doctors when something is off-track. Such features enhance our proactiveness in care, as treatment plans are adjusted accordingly before minor, manageable issues deteriorate into more serious conditions. This proactive approach is what makes AI so transformative in clinical settings.

**Interviewer:** That’s an important point. From your perspective, what are some of the challenges associated with implementing AI and telemedicine?

**Participant P.7:** One of the biggest challenges is the implementation costs. AI-enabled healthcare systems, especially those integrated with telemedicine, can overwhelm smaller institutions that lack adequate financial resources. The upfront investment in infrastructure, training, and system integration is significant, and many facilities struggle to meet these demands.

**Interviewer:** Besides cost, are there any other barriers you foresee in adopting these technologies effectively in the UAE?

**Participant P.7:** Absolutely. Implementing AI and telemedicine in the UAE will automatically result in the creation of huge amounts of data. I don’t think our healthcare system is fully prepared to handle and utilize such volumes of data effectively. AI thrives on data, but if we cannot use that data to make clinical decisions, then the technology will not serve its purpose. Major institutional preparation and infrastructural development are needed to ensure that these technologies benefit both patients and doctors.

**Interviewer:** That’s a valid concern. How do you think these technologies could be better integrated into daily practice?

**Participant P.7:** It’s not just about having the technology; it’s about making sure it fits into our daily practice. If using AI or telemedicine adds more steps or slows us down, it becomes more of a hindrance than a help. The system must be streamlined and user-friendly to truly enhance efficiency and reduce the burden on healthcare providers.

**Interviewer:** Based on your experience, what steps can be taken to ensure that these technologies are more effective?

**Participant P.7:** For one, institutions must invest in robust data infrastructure and training programs for staff to maximize the potential of these tools. Additionally, involving healthcare professionals in the design and implementation processes can ensure that these systems are practical and address real-world needs. Finally, raising awareness among patients about the benefits of these technologies is also crucial to foster acceptance and trust.

**Interviewer:** Thank you for sharing these detailed insights. Is there anything else you’d like to add?

**Participant P.7:** Just that while these technologies hold great promise, we need to approach their implementation thoughtfully. It’s not just about adopting the latest tools but ensuring they truly enhance the care we deliver.

### Interview Transcript: Participant P.8

**Interviewer:** Thank you for joining this study. Let’s begin with your perspective on how AI impacts healthcare delivery.

**Participant P.8:** AI has the potential to handle a lot of the routine, time-consuming tasks that don’t necessarily need a human touch. For example, processing patient records, analyzing patterns, or flagging anomalies in data can all be automated. This frees up time for our doctors and nurses, allowing them to focus on what truly matters—providing personalized and attentive care to patients with complicated cases.

**Interviewer:** That’s an interesting perspective. Do you think AI fully replaces the need for human involvement in certain areas of healthcare?

**Participant P.8:** Not entirely. While AI is excellent for administrative efficiency and supporting clinical decision-making, it’s not a substitute for the human connection in healthcare. Patients often rely on the empathy and emotional support that only humans can provide. So, AI should be seen as a tool to enhance, not replace, human involvement in healthcare.

**Interviewer:** Based on your experience, what are the potential benefits of using AI in healthcare settings?

**Participant P.8:** AI is particularly beneficial in improving efficiency. By automating repetitive tasks, it reduces the workload for healthcare providers, which can help address staff burnout—a significant issue in our field. Moreover, the ability of AI to identify patterns in data can lead to earlier interventions and better patient outcomes.

**Interviewer:** What challenges do you foresee in adopting AI on a larger scale in healthcare?

**Participant P.8:** One major challenge is the initial investment required to integrate AI systems into our healthcare infrastructure. Smaller facilities may find it difficult to allocate funds for these technologies. Additionally, training staff to use AI effectively can be time-consuming and requires significant effort. Another challenge is the skepticism some healthcare professionals have regarding AI’s reliability and its impact on job security.

**Interviewer:** What steps can be taken to ensure AI is integrated effectively?

**Participant P.8:** It starts with awareness and education. Healthcare professionals need to be educated on how AI can support their work rather than replace them. Institutions must also involve clinicians in the design and implementation process to ensure the tools meet real-world needs. Lastly, addressing data privacy and security concerns is critical to gaining the trust of both patients and providers.

**Interviewer:** Thank you for these insights. Is there anything else you’d like to add?

**Participant P.8:** Just that we must approach AI adoption strategically. It’s a powerful tool, but its success depends on how well we align its capabilities with the needs of both patients and providers. If implemented thoughtfully, AI can truly revolutionize healthcare delivery.

### Interview Transcript: Participant P.9

**Interviewer:** Thank you for taking the time to participate in this study. To begin, could you share your thoughts on the role of AI in healthcare?

**Participant P.9:** AI is beneficial and will be beneficial in the future of the healthcare sector. However, its success depends on certain factors. One of the most critical aspects is ensuring the protection of patients' privacy. Many patients are still skeptical about how their data is handled, and without trust, the adoption of AI will face challenges. Additionally, AI should complement human doctors rather than replace them. Patients often feel uneasy about being treated by a programmed machine, so a balance is necessary to maintain the human connection in healthcare.

**Interviewer:** That’s a valuable point. What other factors do you think could encourage innovation and the adoption of technologies like AI in healthcare?

**Participant P.9:** Recognition and appreciation for innovations are crucial. These are non-monetary incentives that are often ignored but are very important in fostering a culture of innovation in healthcare settings. When healthcare professionals and teams feel acknowledged for their efforts to adopt or create innovative solutions, they become more motivated to explore and implement new technologies.

**Interviewer:** How can healthcare organizations address the concerns you’ve mentioned, such as privacy and the balance between AI and human interaction?

**Participant P.9:** Transparency is key. Healthcare organizations need to communicate clearly about how patient data is used and protected. Implementing robust security measures will also build trust among patients. On the human interaction front, training programs can help doctors and nurses understand how to integrate AI into their work without losing the personal touch that patients value. It’s about creating a partnership between technology and human expertise.

**Interviewer:** In your opinion, what steps should be taken to ensure that innovations like AI are sustainable in the long term?

**Participant P.9:** Besides addressing privacy and human interaction, healthcare organizations should focus on creating an ecosystem that encourages continuous improvement. This includes regular updates to AI systems based on user feedback and advancements in technology. Also, governments and policymakers must support innovation through regulations that promote ethical AI use while minimizing barriers to adoption.

**Interviewer:** Thank you for sharing your thoughts. Is there anything else you’d like to add?

**Participant P.9:** Just that the adoption of AI should always be patient-centered. If we can ensure that patients feel secure, valued, and heard, then the integration of AI into healthcare can achieve its full potential.

### Interview Transcript: Participant P.10

**Interviewer:** Thank you for joining us today. To start, can you share your perspective on the role of telemedicine in your clinical practice?

**Participant P.10:** Telemedicine has become an essential tool, especially for our elderly patients. Sometimes mobility is a major challenge for them, as many need regular monitoring and care. Telemedicine helps us connect with these patients more often, allowing us to identify health issues before they escalate, which in turn reduces disruptions to their daily lives. It offers a lifeline, particularly for those who would struggle to make frequent visits to healthcare facilities.

**Interviewer:** That’s insightful. What do you think are the biggest barriers to implementing AI and telemedicine in healthcare settings?

**Participant P.10:** There are quite a few challenges. One major concern is safety. AI algorithms are designed by humans, and unfortunately, human biases can creep into these systems. Such biases could lead to life-threatening errors, which is unacceptable in a clinical setting. I think we still have a long way to go before AI is fully implemented in healthcare. Issues like safety and data privacy must be addressed comprehensively to earn the trust of both doctors and patients.

Another issue is financial. Many insurers don’t cover telemedicine services, leaving patients to pay out of pocket. This can be a huge burden, particularly for those who are already struggling financially. On top of that, some healthcare institutions lack the financial and technical capacity to integrate these technologies, which exacerbates inequalities in healthcare access.

**Interviewer:** That’s an important point. Do you think there are solutions to overcome these barriers?

**Participant P.10:** Absolutely. First, we need more training for healthcare professionals. The lack of training often leads to underutilization of these technologies, which limits their potential to improve the quality of care. Bridging this gap is crucial.

Additionally, incentives play a vital role. Users—whether they are patients or medical professionals—need to feel supported. For example, governments could provide financial support or other incentives to encourage adoption. This would go a long way in addressing the resistance to change that many healthcare professionals feel. Some of my colleagues believe that AI and telemedicine disrupt their usual practices and routines, and some even fear that it takes away their roles and value as medical practitioners.

**Interviewer:** That’s an interesting observation. How do you think these technologies can be better operationalized, particularly in the UAE?

**Participant P.10:** The UAE has made remarkable strides in integrating emerging technologies like AI and telemedicine, but the operationalization of these systems still faces hurdles. For instance, many healthcare systems grapple with insufficient capacity and a lack of standardized systems. This inconsistency creates challenges for the seamless integration of these technologies. Addressing these gaps will ensure that AI and telemedicine benefit everyone, not just patients in well-funded institutions.

**Interviewer:** Thank you for sharing your insights. Is there anything else you’d like to add?

**Participant P.10:** Just that the integration of AI and telemedicine needs to be patient-centered. Beyond the technology, it’s about ensuring that the tools are accessible, safe, and efficient for all stakeholders involved.

### Interview Transcript: Participant P.11

**Interviewer:** Thank you for taking the time to share your insights with us. To begin, how do you think telemedicine impacts the relationship between healthcare professionals and patients?

**Participant P.11:** Telemedicine has its benefits, but it can negatively impact the relationship between healthcare professionals and patients. The lack of physical face-to-face interactions creates room for misinformation and even misdiagnosis in some cases. For example, certain critical details can only be observed during a physical examination, and without those, the clinical decision-making process could be compromised.

**Interviewer:** That’s an important concern. Do you think telemedicine is appropriate for all situations, or are there instances where it might fall short?

**Participant P.11:** Absolutely, telemedicine is not suitable for every scenario. For instance, emergency situations require prompt, in-person evaluations. The lack of physical observation in telemedicine could result in the omission of critical details that inform treatment decisions, potentially leading to incorrect diagnoses or ineffective interventions.

**Interviewer:** That’s a valid point. Do you have any suggestions for addressing resistance to digital health technologies like telemedicine and AI among healthcare professionals?

**Participant P.11:** Yes, I believe resistance can be mitigated through the creation of a dedicated department that focuses on training, raising awareness, and supporting healthcare professionals. This department could adequately prepare us to face such changes. It’s not just about handing over the technology but ensuring that users feel confident and well-equipped to integrate it into their practices.

**Interviewer:** Interesting. How do you feel about being involved in the development process for these technologies?

**Participant P.11:** Our inclusion in the development process makes us feel like partners and co-creators rather than passive users. This partnership leads to systems that align with our unique practices, needs, values, and beliefs. When we feel a sense of ownership over the process and the final product, it’s much easier to adopt and integrate the technology effectively.

**Interviewer:** Those are compelling insights. Any final thoughts you’d like to share?

**Participant P.11:** Yes. While digital health technologies are undeniably beneficial, it’s essential to remember that they need to complement—not replace—core aspects of healthcare delivery, such as trust, face-to-face interactions, and clinical accuracy.

### Interview Transcript: Participant P.12

**Interviewer:** Thank you for joining us today. Let’s begin with your thoughts on how AI technologies impact the relationship between doctors and patients.

**Participant P.12:** Sure. From a patient’s perspective, one of the most reassuring things is knowing that your doctor understands your medical history without you having to repeat it every time. It can be frustrating, especially when you’ve forgotten some details that might be important for making clinical decisions. AI plays a huge role here. It allows doctors to quickly retrieve patient records and even recommend treatments based on those records. This not only builds trust but also makes patients feel more confident in the quality of care they’re receiving.

**Interviewer:** That makes sense. Would you say this has also helped improve the overall patient experience?

**Participant P.12:** Absolutely. The efficiency AI brings is incredible. For example, scheduling systems powered by AI allocate consultation times based on the patient’s specific health issues. This means doctors have enough time to address each patient’s needs thoroughly. It also helps in managing queues—patients know exactly when they’ll see the doctor, which reduces waiting times and frustration. These improvements enhance the overall experience for patients and make healthcare delivery smoother.

**Interviewer:** Beyond operational efficiency, are there any other areas where you see AI making an impact?

**Participant P.12:** Yes, AI can streamline many routine tasks that used to take up a lot of time, such as organizing records or generating reports. This gives healthcare professionals more time to focus on providing personalized care to patients with more complex conditions. But it’s also crucial to note that these systems need to be well-integrated into the daily workflow. If they’re not intuitive or add unnecessary steps, they can actually slow things down, which defeats the purpose.

**Interviewer:** That’s a great point. How do you think these technologies could be optimized further?

**Participant P.12:** Optimization starts with understanding user feedback—both from patients and healthcare providers. These systems need to be updated regularly to adapt to the evolving needs of users. Training is also critical. If the staff doesn’t fully understand how to use AI tools, then the technology won’t reach its full potential. I also believe a balance is necessary. While technology brings efficiency, we shouldn’t lose sight of the importance of human interaction in healthcare. Patients value empathy and personalized attention, and that shouldn’t be overshadowed by digital tools.

**Interviewer:** Thank you for your insights. Is there anything else you’d like to share?

**Participant P.12:** Just that as we move forward, we should focus on building a healthcare system that combines the best of both worlds—technological efficiency and human compassion. That’s how we can truly improve patient outcomes and satisfaction.

### Interview Transcript: Participant P.13

**Interviewer:** Thank you for participating in this interview. Let’s start with telemedicine. How has it impacted your work?

**Participant P.13:** Telemedicine has truly been a revelation for me. It’s not just about convenience; it has improved my ability to make accurate diagnoses and determine the most effective ways to manage different cases. Collaborating with patients through telemedicine has also enhanced my communication and interpersonal skills. Beyond that, it provides a cost-effective mechanism for analyzing large amounts of data in real time, which is invaluable compared to traditional methods.

**Interviewer:** That’s fascinating. How do you feel about the role of telemedicine in managing transmissible diseases?

**Participant P.13:** I strongly support the integration of telemedicine and AI for this very reason. These technologies allow patients to receive care from the comfort of their homes, which significantly reduces the risk of exposure to infectious diseases in crowded hospital settings. It’s an effective way to control the spread of transmissible diseases, especially during pandemics.

**Interviewer:** What about managing chronic illnesses or enhancing community health?

**Participant P.13:** Telemedicine is incredibly effective in this regard. It eases interaction between patients and primary healthcare providers, making it easier to monitor individuals with chronic illnesses. This, in turn, promotes community health and improves patients' quality of life. It’s a win-win situation for both healthcare providers and patients.

**Interviewer:** Let’s discuss AI. Do you think AI has the potential to replace human judgment in healthcare?

**Participant P.13:** No, I don’t think so. AI is excellent at handling routine tasks, but it can never replace the need for clinical judgment. If the algorithm is trained on flawed or incomplete data, it can result in terribly flawed diagnoses. That’s a significant risk that no physician would want to take, particularly when dealing with complex cases that require a personalized approach. AI should complement, not replace, human expertise.

**Interviewer:** That’s an important point. Are there any limitations of telemedicine you’d like to highlight?

**Participant P.13:** Absolutely. Not all medical procedures are suited for telemedicine. Some needs, especially life-threatening ones, require the physical presence of a healthcare professional. For instance, how could procedures like surgery or resuscitation be conducted through a screen? These limitations need to be carefully addressed in discussions about telemedicine’s role in healthcare.

**Interviewer:** That makes sense. What about the human resources aspect?

**Participant P.13:** The widespread adoption of AI and telemedicine in the UAE will inevitably increase the demand for tech-savvy healthcare professionals. They are central to the implementation and effective use of these technologies. Unfortunately, many healthcare workers don’t have enough time outside their work schedules to engage in training. To address this, training programs must be designed with flexible schedules to accommodate their routines while ensuring they gain the necessary knowledge to operate these systems effectively. Otherwise, there’s a risk of errors, which could have serious consequences for patients.

**Interviewer:** That’s a critical observation. Is there anything else you’d like to add before we conclude?

**Participant P.13:** I’d just like to emphasize the importance of finding the right balance. While technology is a powerful tool, it must work in harmony with human expertise to truly benefit both patients and healthcare providers. We need thoughtful integration to make the most of these advancements without compromising the quality of care.

### Interview Transcript: Participant P.14

**Interviewer:** Thank you for joining us today. To begin, could you share your thoughts on the implementation of AI and telemedicine in the UAE healthcare system?

**Participant P.14:** Thank you. AI and telemedicine are promising innovations that have the potential to reshape how we deliver healthcare. However, I’ve observed that these technologies are often constrained by strict regulations. While these regulations are intended to safeguard patient safety and data, they can also hinder full adoption. For instance, certain processes are delayed or restricted because of compliance requirements, which slows down the integration of AI and telemedicine into daily healthcare practices.

**Interviewer:** That’s an interesting point. Could you elaborate on how these regulations impact the efficiency of healthcare delivery?

**Participant P.14:** Sure. While regulations are vital to ensuring patient safety, they sometimes add layers of bureaucracy that slow down the adoption process. For example, approval processes for new AI-based systems can take months or even years, even if the technology has already proven its effectiveness elsewhere. This delay can frustrate healthcare providers and limit our ability to use these tools to their fullest capacity. In some cases, the regulations are so stringent that they make it nearly impossible to customize systems to our unique institutional needs.

**Interviewer:** That’s certainly a challenge. Do you think these regulatory hurdles discourage healthcare professionals from adopting these technologies?

**Participant P.14:** Absolutely. When healthcare professionals see that adopting new technologies involves navigating complex regulations and jumping through numerous hoops, they may feel discouraged. This is particularly true for smaller institutions that lack the resources to handle such administrative burdens. It’s one thing to embrace innovation, but if the process is too cumbersome, even the most forward-thinking institutions may hesitate.

**Interviewer:** Beyond regulations, what other factors do you think influence the adoption of AI and telemedicine?

**Participant P.14:** Training is a fundamental component. If healthcare professionals are not adequately trained on how to use these technologies, they are less likely to adopt them. Training helps remove doubts and misconceptions, builds confidence, and makes us feel competent in using new systems. For example, I’ve seen colleagues become much more open to AI and telemedicine after attending hands-on workshops and seeing the practical benefits. Training also helps bridge the gap between the theoretical potential of these technologies and their actual application in clinical settings.

**Interviewer:** That’s an excellent point. How would you suggest training programs be designed to be most effective?

**Participant P.14:** Training programs should be tailored to the needs of healthcare professionals. They should be practical, interactive, and easy to integrate into our schedules. For instance, offering online modules that can be completed during downtime at work would be a great start. These programs should also include real-world case studies to demonstrate how AI and telemedicine can solve specific problems we encounter daily. Additionally, it’s important to involve senior clinicians in the training process because their endorsement can influence others to adopt these technologies.

**Interviewer:** You’ve mentioned that training builds confidence. Do you think this confidence extends to patient interactions?

**Participant P.14:** Definitely. When healthcare professionals are confident in using AI and telemedicine, it reflects in their interactions with patients. Patients pick up on this confidence and are more likely to trust the technology. Conversely, if a clinician is hesitant or unsure, it can make patients skeptical about the benefits of these innovations. That’s why training is not just about technical skills; it’s also about building trust and communication skills.

**Interviewer:** What about patients? Do you think they face similar challenges in adopting these technologies?

**Participant P.14:** Yes, patients need to be educated about how these technologies work and how they can benefit from them. Many patients are resistant to change simply because they don’t understand it. For instance, telemedicine might seem impersonal to some, but if they’re shown how it can save time and offer convenience, they may be more willing to try it. Awareness campaigns that explain the benefits in simple, relatable terms could go a long way in increasing patient acceptance.

**Interviewer:** That’s a great suggestion. Lastly, what are your thoughts on the long-term outlook for AI and telemedicine in the UAE?

**Participant P.14:** I’m optimistic. The UAE has always been a leader in adopting new technologies, and I believe we can overcome these challenges with the right strategies. This includes revisiting regulations to make them more flexible, investing in comprehensive training programs, and ensuring that both patients and healthcare professionals are on board. If we can address these barriers, I see no reason why AI and telemedicine can’t become integral parts of our healthcare system.

**Interviewer:** Thank you so much for sharing your insights. Is there anything else you’d like to add before we conclude?

**Participant P.14:** Just that while technology is critical, it should never replace the human element in healthcare. AI and telemedicine should be tools that enhance what we do, not substitutes for the personal touch that patients value so much. As we move forward, it’s essential to strike a balance between innovation and the timeless principles of patient care.

### Interview Transcript: Participant P.15

**Interviewer:** Thank you for joining us today. Let’s begin by discussing your overall impressions of AI and telemedicine in healthcare.

**Participant P.15:** AI and telemedicine have undoubtedly revolutionized how we approach patient care. They have streamlined processes, made care more accessible, and improved diagnostic accuracy in many cases. However, alongside these benefits, there are significant concerns, especially regarding data privacy. Patients’ personal data can easily be at risk of being accessed by unauthorized individuals due to a lack of strict measures and standards governing how data managed via AI tools should be stored and protected. This risk makes many patients—and even healthcare providers—hesitant to fully trust these technologies.

**Interviewer:** Data security is a critical issue. In your view, what measures could mitigate these risks?

**Participant P.15:** A robust data management framework is absolutely essential. This framework should clearly define who can access patient data, how it is stored, and the protocols for transmission. Strict encryption standards must be enforced to ensure that sensitive information cannot be intercepted or misused. Moreover, regular audits and monitoring systems should be established to ensure compliance with these policies. Transparency is also important; healthcare institutions should openly communicate how they are safeguarding patient data.

**Interviewer:** That makes sense. How do you think healthcare professionals and patients can be reassured about the safety of their data?

**Participant P.15:** Education plays a key role here. Both patients and healthcare professionals need to understand the measures in place to protect their data. For professionals, comprehensive training should be provided, covering not only how to use AI and telemedicine tools but also the importance of following data security protocols. For patients, simple and clear explanations about how their information is handled and protected can go a long way in building trust. Additionally, involving patients in the conversation about data security could make them feel more confident and valued.

**Interviewer:** Do you believe these concerns significantly hinder the adoption of AI and telemedicine?

**Participant P.15:** Absolutely. If patients feel that their personal information is vulnerable, they are much less likely to embrace these technologies, regardless of the potential benefits. For healthcare professionals, the ethical responsibility of protecting patient data can also create resistance. Many clinicians worry about being held accountable for breaches that might occur due to technological failures. Addressing these concerns isn’t just a technical challenge; it’s fundamental to ensuring widespread adoption and effective use of AI and telemedicine.

**Interviewer:** Aside from data security, are there any other barriers you’ve noticed?

**Participant P.15:** Another issue is the lack of uniform standards across different institutions. Without clear guidelines, there’s a lot of variability in how AI and telemedicine are implemented and used. This inconsistency can create confusion and inefficiencies, further discouraging adoption. Additionally, the high costs associated with implementing these technologies pose a barrier, especially for smaller healthcare facilities. If financial incentives or subsidies were provided, more institutions might be willing to invest in these systems.

**Interviewer:** That’s an important point. Do you think these challenges outweigh the benefits, or can they be overcome?

**Participant P.15:** They can definitely be overcome, but it will require a collaborative effort. Governments, healthcare institutions, and technology developers need to work together to establish standardized protocols, provide financial support, and ensure that these technologies are accessible to all. Without a unified approach, the full potential of AI and telemedicine will remain untapped.

**Interviewer:** How do you see the future of AI and telemedicine in the UAE?

**Participant P.15:** The UAE has made remarkable strides in integrating advanced technologies into its healthcare system, but there’s still work to be done. For AI and telemedicine to reach their full potential, we need to address these barriers comprehensively. This includes not only enhancing data security but also fostering a culture of trust and collaboration among all stakeholders. With the right policies and initiatives, I believe these technologies can transform healthcare delivery in the UAE and serve as a model for other countries.

**Interviewer:** Before we conclude, is there anything else you’d like to add?

**Participant P.15:** Just that while technology can greatly enhance healthcare, it’s crucial not to lose sight of the human element. Patients value the personal connection with their healthcare providers, and no amount of innovation should replace that. The goal should always be to use these tools to support—not replace—the human touch in healthcare.
