# Supplementary material for: Navigating digital frontiers in UAE healthcare: A qualitative exploration of healthcare professionals’ and patients’ experiences with AI and telemedicine
Source: PLOS Digit Health. 2025 Apr 8;4(4):e0000586. doi: 10.1371/journal.pdig.0000586 (PMC11991283; doi:10.1371/journal.pdig.0000586)
Supplement: Attachment — Submitted filename: PDIG-D-24-00287_reviewer.pdf [file pdig.0000586.s002.pdf]

PLOS Digital Health

Navigating Digital Frontiers in Healthcare: A Qualitative Exploration of Healthcare Professionals' and Patients' Experiences with AI and Telemedicine

--Manuscript Draft--

**Manuscript Number:**	PDIG-D-24-00287
**Article Type:**	Research Article
**Full Title:**	Navigating Digital Frontiers in Healthcare: A Qualitative Exploration of Healthcare Professionals' and Patients' Experiences with AI and Telemedicine
**Short Title:**	Exploring AI and Telemedicine: Healthcare Professionals' and Patients' Experiences
**Corresponding Author:**	Azza Alkaabi, Ph.D<br>Rabdan Academy<br>Abu Dhabi, UNITED ARAB EMIRATES
**Order of Authors:**	Azza Alkaabi, Ph.D
	Deena Elsori
**Keywords:**	Artificial Intelligence; Telemedicine; Digital Health Technologies; Healthcare Professionals' Perceptions; Patient Experiences; Healthcare Management; Qualitative Research; Barriers and Facilitators; UAE Healthcare; Remote Healthcare Services
**Abstract:**	Digital technologies have revolutionized many sectors, and healthcare is no exception. Their incorporation into healthcare systems has made the delivery of services increasingly patient-centered, improved the patient-doctor relationship, and removed geographical barriers to accessing medical services. Among the technologies making headlines are AI and telemedicine, the implementation of which has created new and complex dynamics in healthcare delivery. These changes suggest the need to get the perspectives of stakeholders regarding the effectiveness of these technologies and the barriers hindering their adoption. As such, the purpose of this study was to explore the contextual factors influencing the acceptance, implementation, and effectiveness of AI and telemedicine from the perspectives of patients and healthcare professionals. A qualitative descriptive research design was used, whereby the participants were selected using a purposive sampling technique. Based on this technique, 15 participants (8 healthcare professionals and seven patients) were recruited. Semi-structured interviews were used to collect data from them and thematic analysis to analyze their responses. The results obtained indicated that AI and telemedicine were associated with several benefits such as improved convenience, cost and time-saving, control of infectious diseases, as well as boosting primary care and chronic illnesses' management, improved doctor-patient relationships, and making service delivery patient-centered. It was also established that there are barriers and facilitators influencing the adoption and effective use of these technologies. Barriers included misinformation and misdiagnosis, safety concerns, skills gaps, infrastructural and technical limitations, and financial constraints. The main facilitators included creating awareness about the technologies, training healthcare professionals on their effective use, incentivizing their use, and engaging stakeholders in the design, development, and implementation processes. Therefore, it was concluded that investing in the facilitating factors, maximizing the benefits, and minimizing the barriers can improve the adoption and effective use of AI and telemedicine in clinical settings.
**Additional Information:**	
**Question**	**Response**
**Government Employee**<br><br>Are you or any of the contributing authors an employee of the United States government?	No - No authors are employees of the U.S. government.

Powered by Editorial Manager® and ProduXion Manager® from Aries Systems Corporation

Manuscripts authored by one or more US Government employees are not copyrighted, but are licensed under a CC0 Public Domain Dedication, which allows unlimited distribution and reuse of the article for any lawful purpose. This is a legal requirement for US Government employees.<br><br>This will be typeset if the manuscript is accepted for publication.	
**Financial Disclosure**<br><br>Enter a financial disclosure statement that describes the sources of funding for the work included in this submission. Review the submission guidelines for detailed requirements. View published research articles from *PLOS Digital Health* for specific examples.<br><br>This statement is required for submission and **will appear in the published article** if the submission is accepted. Please make sure it is accurate.<br><br>**Funded studies**<br>Enter a statement with the following details:<br>• Initials of the authors who received each award<br>• Grant numbers awarded to each author<br>• The full name of each funder<br>• URL of each funder website<br>• Did the sponsors or funders play any role in the study design, data collection and analysis, decision to publish, or preparation of the manuscript?<br><br>Did you receive funding for this work?	The author(s) received no specific funding for this work.
**Competing Interests**<br><br>On behalf of all authors, disclose any competing interests that could be perceived to bias this work.	On behalf of all authors, we declare that there are no competing interests. No financial, professional, or personal interests could be perceived to bias this work. The authors did not receive any funding for this project, and there are no other relationships or activities that could influence the results or interpretation of our study.

Powered by Editorial Manager® and ProduXion Manager® from Aries Systems Corporation

This statement will be typeset if the manuscript is accepted for publication.<br><br>Review the instructions link below and PLOS Digital Health's competing interests policy to determine what information must be disclosed at submission.	
**Data Availability**<br><br>Provide a **Data Availability Statement** in the box below. This statement should detail where the data used in this submission can be accessed. This statement will be typeset if the manuscript is accepted for publication.<br><br>Before publication, authors are required to make all data underlying their findings fully available, without restriction. Review our PLOS Data Policy page for detailed information on this policy. Instructions for writing your Data Availability statement can be accessed via the Instructions link below.	All relevant data underlying the findings of this study are available within the manuscript and its Supporting Information files. The anonymized interview transcripts and thematic analysis data are stored in a secure repository to ensure participant confidentiality. Data can be accessed upon reasonable request by contacting the corresponding author, provided that the request complies with ethical guidelines and participant consent.

Powered by Editorial Manager® and ProduXion Manager® from Aries Systems Corporation

Manuscript

Click here to access/download;Manuscript;Effectiveness of Digital Health Technologies.pdf

RESEARCH ARTICLE

Navigating Digital Frontiers in Healthcare: A Qualitative Exploration of Healthcare Professionals' and Patients' Experiences with AI and Telemedicine

Authors

Azza Alkaabi

aalkaabi@ra.ac.ae

ORCID ID: 0009-0000-3827-1808

Deena Elsori: delsori@ra.ac.ae

ORCID ID: 0000-0002-1063-5101

Abstract

Digital technologies have revolutionized many sectors, and healthcare is no exception. Their incorporation into healthcare systems has made the delivery of services increasingly patient-centered, improved the patient-doctor relationship, and removed geographical barriers to accessing medical services. Among the technologies making headlines are AI and telemedicine, the implementation of which has created new and complex dynamics in healthcare delivery. These changes suggest the need to get the perspectives of stakeholders regarding the effectiveness of these technologies and the barriers hindering their adoption. As such, the purpose of this study was to explore the contextual factors influencing the acceptance, implementation, and effectiveness of AI and telemedicine from the perspectives of patients and healthcare professionals. A qualitative descriptive research design was used, whereby the participants were selected using a purposive sampling technique. Based on this technique, 15 participants (8 healthcare professionals and seven patients) were recruited. Semi-structured interviews were used to collect data from them and thematic analysis to analyze their responses. The results obtained indicated that AI and telemedicine were associated with several benefits such as improved convenience, cost and time-saving, control of infectious diseases, as well as boosting primary care and chronic illnesses' management, improved doctor-patient relationships, and making service delivery patient-centered. It was also established that there are barriers and facilitators influencing the adoption and effective use of these technologies. Barriers included misinformation and misdiagnosis, safety concerns, skills gaps, infrastructural and technical limitations, and financial constraints. The main facilitators included creating awareness about the technologies, training healthcare professionals on their effective use, incentivizing their use, and engaging stakeholders in the design, development, and implementation processes. Therefore, it was concluded that investing in the facilitating factors,

maximizing the benefits, and minimizing the barriers can improve the adoption and effective use of AI and telemedicine in clinical settings.

Navigating Digital Frontiers in Healthcare: A Qualitative Exploration of Healthcare Professionals' and Patients' Experiences with AI and Telemedicine

Introduction

The rapid advancement in digital technologies over the past few decades has revolutionized many sectors, with healthcare being no exception. These technologies have pushed healthcare delivery to new levels by making it increasingly patient-centered and improving the patient-doctor relationship [1]. They have also brought significant ideological and structural transformation to healthcare provision and management, including records maintenance and medication [1]. Today, patients do not have to travel from their homes to have face-to-face interactions with physicians because of the emergence of telemedicine services, which involves using information and communication technologies (ICT) to provide or support healthcare [2]. This observation suggests that it is now possible for patients to get health services without being in the same geographical location as the providers.

Innovations, such as Artificial Intelligence (AI), have significantly transformed healthcare delivery by improving the accuracy of diagnosis and treatment and allowing healthcare providers to offer their services remotely [2]. As such, the traditional geographical barriers that limited healthcare delivery are now being broken, making it possible for people in remote and underdeveloped areas to get services. The analytic capabilities of AI also mean that enormous amounts of data can be analyzed with relative ease, thus facilitating data-driven or knowledge-based decision-making processes [3]. While there are still challenges in its implementation, the technology's prospect of transforming the healthcare sector is immense and unquestionable. It provides a basis for enhancing the affordability, quality, and accessibility of healthcare services [3].

AI and telemedicine also provide an opportunity through which medical knowledge can be democratized through improved access to health-related information and data, allowing patients to make informed decisions regarding their health. It is worth noting that the concept of electronic health (e-health) arose after personal computers boomed in the 1990s [1]. These computers paved the way for the development of networks upon which telemedicine could be offered. They also provided the foundation for the emergence of other innovations, such as social media platforms and smartphones, which have contributed to the development of mobile health [1]. Consequently, patients and healthcare providers can equally access data and information that allow them to make collective decisions for the betterment of patients' care.

Against this background, it can be argued that digital health technologies represent not just a technological transformation but also a fundamental restructuring of healthcare systems, including how a patient relates to the doctor and how treatment is performed. They represent a paradigm shift in how healthcare is provided, including making the services more patient-centered [1]. Medicines are also becoming increasingly individualized or personalized instead of being generalized. Furthermore, the traditional hierarchical model has been replaced with a more collaborative model, leading to improved partnership between all the stakeholders involved, especially in information dissemination and sharing [1]. Digitalization started and later facilitated the introduction of e-patients, which has contributed significantly to the empowerment of patients through improved access to health information/knowledge. It has also provided a mechanism for them to interact with their fellow patients and share experiences [1].

Since healthcare providers are critical in the integration of these digital health technologies, their views concerning the opportunities and challenges they present are vital. To them, the transition towards digitalization has created new and complex dynamics in healthcare delivery,

considering that patients are gradually appreciating the benefits of such interventions as AI and telemedicine, especially in areas or situations where physical interactions are infeasible. These technologies have not only encouraged proactivity in healthcare delivery but also mitigated the stresses associated with frequent visits to hospitals [4]. However, like any other technological intervention, this change has not been free of challenges, most of which are associated with concerns about data privacy violations [4]. This observation suggests the need to understand the contextual factors (barriers and facilitators) affecting digital health technologies' acceptance, implementation, and effectiveness. A comprehensive understanding of these factors is crucial because it can provide the basis from which the strengths/facilitators could be maximized and the weaknesses/barriers mitigated.

Although the available literature has provided a glimpse of the factors influencing the adoption of digital technologies, it is evident that a comprehensive investigation of the dynamics involved is needed. The present study sought to bridge this gap by exploring the contextual factors influencing the acceptance, implementation, and effectiveness of AI and telemedicine from the perspectives of patients and healthcare professionals. Its rationales included the need to bring new insights into how these technologies are transforming the healthcare sector, particularly the delivery of services to patients. It also sheds light on the strategies that can be adopted to improve the acceptability and effectiveness of AI and telemedicine in addressing modern healthcare challenges. Besides these contributions, the knowledge gained from this study can provide the foundation for enacting policies to boost effectiveness and make the implementation of these technologies seamless. It sought to fulfill three objectives: (1) explore healthcare professionals' perceptions and experiences with digital health technologies, focusing on AI and telemedicine; (2) identify barriers and facilitators affecting the adoption and effective use of AI and telemedicine in

clinical settings; and (3) understand patient experiences and satisfaction levels with AI and telemedicine interventions.

Methods

Study Design

The primary purpose of this study was to explore the contextual factors influencing the acceptance, implementation, and effectiveness of AI and telemedicine from the perspectives and experiences of patients and healthcare professionals. To this end, it relied on a qualitative descriptive research design, which describes phenomena from the participants' perspectives, language, and experiences. As Doyle et al. [5] observe, this research design is primarily used in studies seeking to reveal the 'who, what, and where' of a specific phenomenon or event based on participants' perspectives. It also provides detailed descriptions of objects based on real-world experiences. These characteristics made it the most suitable design for the present study, considering that it sought to reveal the participants' views concerning the factors influencing the adoption of digital health technologies.

Sampling Procedure and Sample Size

Based on the above research design and the study's objectives, a purposive sampling technique was used to select the participants. The rationale for choosing this technique was the finding that most qualitative studies align themselves with the non-random sampling. As Peterson [6] observed, the data sources for a descriptive qualitative study are selected purposively, whereby specific considerations must be made before choosing a source. He defined purpose sampling as a recruitment method in which study subjects are chosen based on specified criteria. In other words, the researcher recruits only knowledgeable and experienced participants in the subject being studied. Since the present study sought to uncover the contextual factors influencing the

effectiveness of AI and telemedicine, it was essential to select the right participants. As such, we were obliged to choose participants with direct experience with both digital health technologies. Several inclusion criteria were used to obtain the desired research population. First, the participants had to be either UAE healthcare professionals (doctors, nurses, and telemedicine specialists) with experience in AI and telemedicine or patients who have interacted with or been served using these digital health technologies in the UAE. Secondly, administrators of health institutions that have adopted and implemented digital health technologies were considered. Another criterion for inclusion was experience as an IT staff in a healthcare institution that has implemented AI and telemedicine.

Since it was practically impossible to collect data from all healthcare professionals in the UAE, the scope was narrowed to hospitals and clinics in Dubai and Abu Dhabi that have incorporated AI and telemedicine. We searched the internet to learn about the hospitals and clinics in the two Emirates that have used or implemented AI and telemedicine services. Several names came up, including Dubai Healthcare Authority (DHA), Mubadala Telemedicine Centre, and Aster DM Healthcare. Their contacts and locations were established online, thus helping us to contact the administrations and ask permission to interview their personnel. To access and recruit the participants, we used referrals and professional networks, whereby the few health professionals identified initially were asked to recommend other people they know who have interacted with or used AI and telemedicine. However, only individuals who satisfied the above criteria were selected and sent invitations via email. Of the 30 invitations sent, only 18 participants agreed to be interviewed, but 3 were dropped because they did not meet the specified criteria. Consequently, only 15 (eight healthcare professionals and seven patients) participated in the interview. This number was deemed enough based on the saturation principle, which holds that there is no need

for additional interviews or data collection if no new insights concerning the research problem are emerging [7]. In other words, when the responses from the interviewees start producing similar themes without variability, the sample size is considered enough. This principle helped us determine that the sample size of 15 was enough for the present study. It was observed that the last three interviewees offered almost similar responses, suggesting no need for further data collection or interviews. Table 1 below shows the participants' demographic characteristics.

Table 1: Demographic characteristics of the participants

Participant Code.	Age	Gender	Background
P.1	35	Male	Patient
P.2	34	Female	Doctor
P.3	40	Female	Patient
P.4	42	Female	Patient
P.5	38	Male	Patient
P.6	50	Male	Nurse
P.7	56	Female	Doctor
P.8	54	Male	Administrator
P.9	45	Male	Patient
P.10	41	Male	Administrator
P.11	54	Male	Telemedicine Specialist
P.12	36	Female	Patient
P.13	39	Male	Doctor
P.14	37	Female	Nurse
P.15	48	Female	Patient

Data Collection Methods

The study relied on semi-structured interviews to collect the necessary data and fulfill the research objectives. As indicated above, the participants comprised healthcare professionals, administrators, patients, and IT staff. The questions asked during the interviews were open-ended and aimed at understanding the participants' experiences, perspectives, and attitudes toward AI and telemedicine. An interview guide that was developed and pre-tested among a small sample of healthcare professionals and patients was used to guide the process. The feedback acquired from

these people helped to modify the questions where necessary to make them effective in fulfilling the objectives. All interviews were scheduled to take at most 60 minutes, with interviewees who were inaccessible for a face-to-face or physical conversation contacted through Skype, Microsoft Teams, and any other applicable online platform.

All the conversations began by asking personal questions geared towards establishing the interviewees' demographic profile. After that, the interview transcript was divided into three sections based on the research objectives. The first section contained questions related to the first objective (exploring healthcare professionals' perceptions and experiences with digital health technologies, focusing on AI and telemedicine). They included the following: (1) how would you describe your experiences with using AI and telemedicine in your practice? (2) What do you see as the main benefits and challenges of these technologies in healthcare? (3) How has the use of AI and telemedicine affected your decision-making, patient interactions, and effectiveness in your practice?

The second section comprised questions related to the second objective, which was about identifying the barriers and facilitators affecting the adoption and effective use of AI and telemedicine in clinical settings. They included the following: (1) what do you think are the primary barriers to the adoption of AI and telemedicine in clinical settings? (2) What facilitating conditions do you think are needed for the effective use of AI and telemedicine? And (3) what advice would you give to an administrator or a healthcare institution seeking to integrate these technologies into their systems? These questions were primarily directed to physicians because they are the ones who directly interact with these technologies in clinical settings. However, the administrators' opinions were also sought because they oversee the adoption and implementation of technologies in healthcare institutions.

The last section contained questions related to the final objective (understanding patient experiences and satisfaction levels with AI and telemedicine interventions). Some of the questions asked included: (1) how would you describe your experience with AI and telemedicine? (2) As a healthcare practitioner, what observations have you made regarding patients' satisfaction with AI and telemedicine? And (3) In what ways do you think AI and telemedicine could be improved to enhance patient experience? These questions were primarily directed to healthcare professionals and patients who have interacted with AI and telemedicine. The reason for seeking the views of healthcare professionals was to understand the feedback they receive from patients regarding these technologies.

Data Analysis

The data obtained from the interviews were analyzed using thematic analysis, which involves identifying themes and patterns from the participants' responses [8]. Codes are used to group the data into themes, highlighting the key insights from the interviews. The primary difference between thematic analysis and other qualitative data analysis methods is that it does not limit the researcher to a specific theory, thus facilitating detailed description and organization of data. As observed by Braun and Clarke [8], thematic analysis is not connected to any theoretical frameworks, meaning that it can work within diverse frameworks. In the present study, codes were developed and organized into themes and sub-themes. These codes helped to highlight the most significant themes based on the number of times they appeared from the participants. A theme was considered significant if it featured in more than half of the responses.

The conceptually similar themes were grouped together before being broken down into different sub-themes and their connections identified. After that, we assigned quotes from the interviewees to their corresponding themes, thus helping to map out patterns. We then used

feedback from the participants to refine the identified themes. Refining and reviewing these themes and sub-themes were necessary to ensure they accurately reflected the interviewees' perspectives. After refining, they were then analyzed further to determine whether there were differences in the participants' views based on their demographic characteristics, including age, gender, experience in using telemedicine, and specialty.

Ethical Considerations

Various ethical considerations were made during the recruitment and data collection processes. The first one involved obtaining ethical permissions/approvals from all relevant authorities before commencing the study. After that, the process of obtaining consent from potential participants kicked off, whereby an informed consent form was sent to them, communicating the research purpose and process. All the individuals contacted were given explanations of what the study entailed and why their inputs/views were needed. They were also reminded that their participation was voluntary, and they could withdraw at any stage without adverse repercussions or victimization. Another measure we took when obtaining consent was to give these people sufficient time to process the information and ask questions to get clarification. After that, those willing to participate were asked to sign the consent form to show that they had voluntarily agreed to participate in the study.

The third ethical consideration involved preserving the anonymity and confidentiality of the interviewees, whereby all the measures were taken to ensure that their privacy and confidentiality rights were not violated. For instance, all the signed consent forms were kept in a safe place where only we (the authors) could access them to protect the participants' personal details from third-party access. The participants were assured that their personal details would not be used for any other purpose apart from what was communicated when obtaining consent. They

were also assured that their names and other identifying details would be concealed when reporting the findings. Although this is digital health research, we ensured that no personally identifying information regarding the patients was recorded due to ethical concerns involved. Any sensitive data that had been recorded was deleted as soon as possible to prevent data breaches that may expose patients' medical history. The participants were also allowed to go through the identified themes and correct any information that could put their privacy at risk.

Validation of Findings

Member checking was used to validate the findings made from the interviews. This technique involved sharing analyzed data/preliminary results with some of the participants to verify that the findings were accurate, trustworthy, and representative of the views expressed by the interviewees. As such, it improved research credibility by allowing the participants to go through analyzed data and correct possible misrepresentations of their opinions or narratives. While it could have been done in focus groups, where more than one participant is involved [9], we contacted the participants individually. The primary reason for using member checking rather than other validation methods was its recognition that the participants are knowledgeable and can play a critical role in data analysis and interpretation [9].

Results

A thematic analysis of participants' responses yielded several themes organized based on the research objectives: 1) healthcare professionals' perceptions and experiences with digital health technologies, focusing on AI and telemedicine; 2) barriers and facilitators affecting the adoption and effective use of AI and telemedicine clinical settings; and 3) patient experiences and satisfaction levels with AI and telemedicine interventions.

Theme 1: Healthcare professionals' perceptions and experiences with digital health technologies, focusing on AI and telemedicine

The first objective of this study was to explore healthcare professionals' perceptions and experiences with digital health technologies, focusing on AI and telemedicine. Various perspectives emerged from the participants' responses and were classified into two broad categories: benefits and drawbacks/challenges.

1.1 Benefits of AI and telemedicine

1.1.1 Enhancing patient participation and facilitating patient-centric care. One of the questions asked when assessing healthcare professionals' perceptions and experiences with AI and telemedicine was about the main benefits and challenges of these technologies in healthcare. This question was motivated by our curiosity about what healthcare professionals think regarding the benefits of AI and telemedicine. The overarching view among the interviewees was that these technologies allow patients to participate more in their treatment and rehabilitation processes. In particular, telemedicine was identified as a crucial technology in this endeavor by providing healthcare professionals with a mechanism through which they can assign responsibilities to patients and engage them in setting goals together. It also allows them to give more intensive care to the patients who require such care because their needs can be assessed more comprehensively.

According to one of the nurses interviewed, *'digital health technologies such as telemedicine have now made it easier for us to transfer some of the responsibilities to the patients. This way, the patients feel they are participating in efforts to improve their well-being, thus boosting their understanding of why specific decisions have been made. It is also an advantage for us and the entire healthcare system because assigning some responsibilities to patients allows us to devote time to those with severe conditions or incapable of taking care of themselves.'* [P.6].

Telemedicine was also attributed to the creation of more informed and proactive patients. All the physicians interviewed insinuated that telemedicine and other digital health technologies allow them to communicate more intensively and resourcefully with their patients, thus helping them to gather vital information even before meeting them physically. This information helps them to understand patients and their needs in a better way. One of the interviewees shared the following:

'Incorporating AI and telemedicine in our facility has allowed us to interact with the patients more and understand their needs. Consequently, the doctor-patient relationship improves, thus increasing the possibility of achieving the desired outcomes. As a doctor, I prefer working with proactive patients who are willing to engage constructively in matters concerning their health. Such patients are easy to handle compared to the passive ones.' [P.2]

1.1.2 Cost and time saving. AI and telemedicine were also associated with cost and time-saving. All the participants, including the patients, observed that using these technologies can save many expenses for the patients and the healthcare system as a whole. The view expressed by most of the participants was that digital health technologies eliminate duplication of roles in healthcare systems, thus reducing the associated inefficiencies and costs. They also ensure that patients are not subjected to unnecessary procedures that make them waste their productive time. One of the interviewees (a doctor) argued that telemedicine has improved his productivity and efficiency and made him a better clinician. He shared the following:

'Telemedicine has been a revelation in my work because it has enhanced my ability to make accurate diagnoses and identify the most effective ways to deal with patients or manage different cases. In particular, my communication and interpersonal skills have improved significantly by collaborating more with patients in the provision of care. These technologies also provide a

mechanism for analyzing large quantities of data in real-time and at a lower cost than the conventional human-centric ways of doing things. Their cost-cutting benefits extend beyond data analysis efficiency to saving patients from the hassles of traveling from their homes to hospitals and enduring challenges such as traffic jams and others.' [P.7].

1.1.3 Controlling the spread of infectious diseases. Another lesson learned from the participants' responses is that using AI and telemedicine can help control the spread of contagious diseases, especially during pandemics such as COVID-19. All the healthcare professionals indicated that transmissible diseases like flu can be controlled using AI and telemedicine, whereby patients are pre-screened for possible infectious illnesses before visiting a healthcare facility. Apart from preventing transmission, such a move could save the sick from having to travel long distances to see a doctor in his working station. One of the nurses observed the following:

'The reason why I support the integration of AI and telemedicine is their potential to help in controlling the spread of transmissible diseases. These technologies make it possible for patients to get care in the comfort of their homes or working stations, thus saving them from the hassles of navigating traffic jams or queues in hospitals, where the likelihood of contracting infectious illnesses such as flu is significantly high. I believe that the more patients are treated from their homes, the less exposure they get to other patients' germs, which is beneficial to everyone involved, particularly the elderly or those suffering from chronic illnesses.' [P.6].

1.1.4 Boosting primary care and chronic illnesses management. Several interviewees also cited AI and telemedicine as one of the interventions that could improve primary care and management of chronic illnesses. They noted that visiting primary care facilities and professionals is vital to the promotion of healthy behaviors, especially at the family level. In this case, AI and telemedicine help to connect the patients with the healthcare professionals and get help without

having to visit the institution for physical interactions. Patients can also use these technologies to book an appointment with the available nurses or doctors, which is essential for those with chronic conditions. One of the doctors explained the following:

'Telemedicine makes it possible for patients to interact regularly with primary care professionals, which is vital in promoting the community's health. People with chronic diseases can also be monitored closely, thus improving their quality of life.' [P.13].

1.2 Challenges/drawbacks of using AI and telemedicine

After learning about the benefits of AI and telemedicine, the next batch of questions related to the challenges faced when using these technologies or the drawbacks. Several challenges were highlighted, including misinformation and misdiagnosis, safety concerns, and financial constraints.

1.2.1 Misinformation and misdiagnosis. Despite the immense potential that AI and telemedicine have to revolutionize the healthcare sector, the participants agreed that using these digital health technologies can be damaging/detrimental, if they are not designed the right way or implemented correctly. They argued that there is a need for more evidence-based clinical assessments to enhance their effectiveness. In particular, the lack of physical/face-to-face interactions was identified as one of the limitations of telemedicine and a weakness that could create room for misinformation. In the words of the telemedicine specialist interviewed in this study:

'Telemedicine is like any other digital technology. When configured and used in the right way, it can bring substantial benefits to the healthcare system. However, when misused, it can have negative impacts, especially on the relationship between the patient and healthcare professionals. For instance, the technology eliminates face-to-face interactions, which are

essential in understanding the needs of patients. When such interactions lack, room for misinformation is created, whereby the parties involved do not get the correct information. This implies that the possibility of misdiagnosing a patient is significantly high.' [P.11].

1.2.2. Safety concerns. The most highlighted drawback of AI and telemedicine was safety concerns, especially when AI is involved. All the participants noted that AI algorithms are not perfect because they are designed by humans, suggesting that they can make substantial errors that endanger patients' lives. The strict regulations under which these technologies operate were also identified as a hindrance. Although they are designed to safeguard patients' safety and enhance data integrity, they can limit the extent to which these technologies are employed or how they are used. According to one of the administrators:

'Safety concerns are the primary barriers hindering the widespread use of AI in clinical settings. The fact that AI algorithms are designed by humans implies that they can be influenced by human biases and cause life-threatening errors. To this end, I would say that AI has a long way to go before it can realize its full potential. It needs to address the issue of safety and data privacy for people to trust it.' [P.10].

1.2.3 Unsuitability for emergency care. Another challenge highlighted by the participants was the unsuitability of these digital health technologies for medical emergencies. It emerged that using telemedicine can lead to delays when administering medication because there are some procedures that doctors cannot carry out remotely. For instance, during a medical emergency, a patient may require life-saving care that only a trained physician can deliver physically. Laboratory tests may also be needed to diagnose the patient. These activities cannot be conducted remotely, thus highlighting the need for further development of these technologies to enhance their suitability for emergency care. As one of the doctors observed:

'While telemedicine can facilitate quick medical attention, there are some emergencies that the technology cannot help. For instance, a life-threatening emergency requires the presence of a doctor or qualified healthcare professional to administer life-saving care. Laboratory tests must also be conducted to determine the root cause of the emergency. These procedures cannot be conducted remotely, meaning the patient must be admitted to the hospital. It is also worth noting that even when emergency care is possible, some patients fail to report a symptom that is crucial in making the correct diagnosis, thus increasing the possibility of misdiagnosis or administering the wrong medications. Some of these symptoms are easy to detect in a face-to-face interaction or in-person care.' [P.13].

1.2.4 Financial constraints. Although AI and telemedicine have been hailed for contributing to cost and time saving, the participants identified their integration into the healthcare system as an expensive endeavor due to the significant investment required in establishing the infrastructure. While these initial costs may not be a primary concern for big institutions, the administrators interviewed in this study indicated that the smaller and resource-constrained ones might find it infeasible, considering that there are other costs to mind, including putting measures in place to mitigate the ethical issues associated with using AI in healthcare. One of the administrators shared the following:

'The cost of implementing AI and telemedicine in healthcare can be prohibitive, especially for smaller institutions with limited resources. Since they are relatively new technologies, a significant amount of money is needed to ensure that they are not only working properly but have also met the standards and are consistent with the existing regulations. Another important issue to highlight is that insurance companies do not cover some telemedicine services, meaning that patients may have to pay from their pockets.' [P.8].

Theme 2: Barriers and facilitators affecting the adoption and effective use of AI and telemedicine clinical settings

The second objective of this study was to establish the factors that influence the adoption and effective use of AI and telemedicine in clinical settings. Based on the participants' responses, several themes were identified and broadly divided into barriers and facilitators.

2.1 Barriers

2.1.1 Dealing with voluminous data and strict regulations. When asked to highlight the primary barriers influencing the adoption and effective use of AI and telemedicine, most of the participants, especially the doctors, indicated dealing with voluminous data and many regulations was one of the hindering blocks. This was a surprising and unexpected finding, considering that AI and digital technologies are primarily developed to help users deal with voluminous data. In fact, some of them insinuated that these technologies could be more beneficial to the patients than the doctors because of the influx of data that the doctors have to deal with. Such data can create new workflows for healthcare providers, who must attend to the patients and address their needs. One of the physicians argued the following:

'I think one of the challenges affecting the implementation of AI and telemedicine in the UAE is that our healthcare system is not fully prepared for the enormous amounts of data from different kinds of patients. There is a need for further development of these technologies to ensure that both the patients and doctors are operating on the same page and they all understand the dynamics involved in their use.' [P.13].

Another issue connected to this barrier is concerns about data protection. All the participants, including the patients, indicated that their greatest fear regarding the incorporation of AI into telemedicine was the potential of data breaches and misuse. They were skeptical about the

ability of the existing security mechanisms to safeguard the privacy of their digitally collected personal data. This skepticism has led to negative assessments of these technologies among some sections of the stakeholders, thus limiting their widespread adoption and effective use. As one of the patients observed:

'These technologies will only be widely accepted if they address the fears associated with data protection. Users need to feel that their digitally collected data is secure and not used for other purposes besides treatment or care provision to the patient. The negative assessment of such technologies as AI and telemedicine is based on the fear that sufficient mechanisms have not been put in place to safeguard patients from breach and misuse. If such mechanisms are developed, I believe the skepticism around these technologies would be addressed.' [P.1].

2.1.2 Infrastructural and technical shortcomings. The participants revealed that despite the country's advanced digital infrastructure, there are still infrastructural and technical barriers that continue to hamper the widespread adoption and effective use of AI and telemedicine in clinical settings. The most frequently mentioned shortcomings included incompatibility with doctors' daily workflows, insufficient capacity among the small institutions, and a lack of standardized systems to be used across the country. One of the administrators shared the following:

'The UAE has made significant progress in expanding the digital infrastructure, but more needs to be done in operationalizing digital health technologies such as AI and telemedicine. In particular, the issue of insufficient capacity and lack of standardized systems must be addressed to make the implementation process seamless. I would suggest that the developers of these technologies involve all the stakeholders, especially the healthcare professionals, to address some of the technical and infrastructural barriers that have hindered their widespread applications.' [P.10].

2.1.3 Internal resistance to change. The participants indicated that the adoption and effective use of AI and telemedicine in clinical settings are also hindered by resistance to change. This barrier was linked to the fact that changes in the working dynamics are inevitable whenever a new technology is introduced. As such, some of the targeted users of the technology oppose its implementation because they would like to maintain the status quo. Many of the healthcare professionals interviewed in this study proposed the establishment of digital transformation departments in healthcare facilities to help in the transition whenever a new technology is introduced. The following sentiments were given by one of the participants:

'Like any other technology, the adoption and effective use of AI and telemedicine have been hindered by resistance to change. People usually perceive change as a disruption to their routine, and these digital health technologies are no exception. Some healthcare providers may see them as unnecessary disruption that could alter their way of doing things or make them lose autonomy in their work. I think this fear of change can be mitigated by creating a digital transformation department to help create awareness about the need for new technologies and provide the necessary conditions for effective implementation.' [P.11].

2.1.4 Skills gap. The shortage of skilled professionals to aid the development, implementation, and maintenance of new technologies was also cited as one of the obstacles hindering the widespread of digital health technologies, particularly the use of AI. The participants noted that for these technologies to be used effectively, healthcare workers and all the stakeholders involved must be equipped with the necessary skills to deal with the resulting changes. In other words, tech-savvy healthcare workers are needed to facilitate the acceptability and use of AI and telemedicine in clinical settings. One of the nurses observed the following:

'The widespread adoption and effective use of AI and telemedicine in the UAE healthcare system will automatically increase the demand for tech-savvy employees, especially healthcare professionals, because they are central to their implementation and use. As such, more training is needed to bridge the existing gap.' [P.13].

2.2 Facilitators

After describing the barriers affecting the adoption and effective use of AI and telemedicine, the participants were asked to highlight the factors they thought could facilitate the adoption of these technologies and their effective use. Several themes were identified from their responses, including the need for awareness of AI and telemedicine, thorough training and education to healthcare professionals, healthcare providers' engagement in the development and implementation of digital health technologies, and incentivizing the use of digital health technologies.

2.2.1 Awareness of AI and telemedicine. The participants cited increased awareness as one of the facilitators of improved acceptability and effective use of AI and telemedicine in the UAE healthcare sector. Most of them suggested that more awareness would help promote an understanding of the benefits of these technologies to not only the clinicians but also the healthcare system as a whole. Once the target users understand their benefits, they would most likely accept their implementation and put more effort into ensuring they are effective. For instance, one of the participants suggested that if healthcare providers learn that AI and telemedicine can improve their effectiveness by reducing their workload, they will most likely adopt them in their workplaces. He explained the following:

'Among the many reasons people resist change is a lack of awareness regarding the benefits of the proposed change. To this end, I believe that increased awareness is one of the key

facilitators of increased adoption and effective use of these technologies. All the stakeholders, including the patients and clinicians, must understand the benefits or usefulness of incorporating AI and telemedicine in clinical settings for them to embrace and accept them.' [P.2].

2.2.2 Thorough training and education to healthcare professionals. All the participants shared the view that training and education are the primary catalysts for the effective use of AI and telemedicine in clinical settings. It emerged that training and educational programs focusing on the effective use of these digital health technologies are critical in encouraging the target users to accept them within their domain of operations. Most participants noted that training increases the feeling of self-efficacy and perceived usefulness of the technology, thus providing incentives for adoption. As observed by one interviewee, *'Training is a critical part of any successful implementation and use of a new technology. It not only improves the target users' feeling of self-efficacy but also improves their understanding of the technology's usefulness to their work. When a person feels they have what it takes to use specific technology to accomplish a task effortlessly, they will inevitably accept its implementation provided it does not pose any substantial threat to their job security.'* [P.14].

Some participants explained the need for healthcare professionals to have flexible schedules that would allow them to attend training. They noted that training is an exercise that requires sufficient attention and time, but these professionals usually lack the spare time needed to learn how to use new technologies. As such, organizations must go beyond creating training programs and provide clinicians with sufficient time to participate in those programs. As one of the interviewees explained:

'Training requires time, and most healthcare workers often lack sufficient time outside their official working hours to engage in such endeavors. To this end, I think it is not enough just to

establish training programs. Healthcare providers require flexible schedules that allow them to learn about AI and telemedicine and how they can incorporate them into their work to boost productivity and effectiveness.' [P.7].

2.2.3 Engaging healthcare providers in the development and implementation of digital health technologies. The participants expressed the need for the primary stakeholders (healthcare professionals) to be involved in developing and implementing AI and telemedicine to boost acceptance and effective use. They noted that involving the target users in developing these technologies helps the developers understand their needs and how they should be addressed. In particular, the developers need to understand how the interfaces should be developed to make them user-friendly and productive. One of the participants indicated the following:

'To mitigate some of the infrastructural and technical barriers associated with using AI and telemedicine in clinical settings, healthcare professionals must be involved in the development and implementation processes. Such a move helps to design and create applications that meet users' needs and expectations, thus boosting their acceptability adoption intentions.' [P.13].

2.2.4 Incentivizing the use of digital health technologies. A significant number of participants also highlighted the use of incentives as a facilitator of the adoption and effective use of AI and telemedicine. They argued that incentives from government and nongovernmental organizations are critical in boosting the effectiveness and acceptability of these technologies in clinical settings. Some of the incentives highlighted included support services and free training for healthcare professionals. One of the participants noted that *'incentives are an indispensable part of the successful implementation and use of new technologies. The users must understand the gains of using a new technology before adopting it, and AI and telemedicine are no different. They also*

need to feel that all the facilitating conditions have been cared for by the relevant authorities.' [P.15].

Theme 3: Patient experiences and satisfaction levels with AI and telemedicine interventions

The last objective of this study was to understand patients' experiences and satisfaction levels with AI and telemedicine. To this end, the patients were asked to describe their experiences with these technologies, while the healthcare professionals were asked to explain what they observed regarding patients' satisfaction with AI and telemedicine. All the participants were also asked to give their recommendations concerning how the technologies can be improved to enhance patient experience. Based on their responses, several sub-themes were identified, including convenience, enhanced frequency and quality of communication, improved patient-doctor relationships, proactivity in care delivery, and quick discharge of patients.

3.1 Convenience. All the patients interviewed in this study associated AI and telemedicine with improved convenience. It emerged that the most significant benefit the patients attach to these technologies is the ability to receive 24-hour care from the comfort of their residences. Most of them argued that AI and telemedicine provide an opportunity for patients to be monitored from their homes without having to experience the challenges of traveling to healthcare facilities to seek medical attention. In other words, they provide a mechanism through which a physically weak person or one who is in a delicate situation can receive medical attention without having to leave their familiar environment. They identified videoconferencing as one of the technologies that have significantly improved convenience by providing an alternative way of meeting a healthcare professional without physical appointments. One of the patients noted the following:

'The most significant benefit I can associate with telemedicine is that it makes the process of seeking healthcare seamless and convenient. With these technologies, my condition can be

monitored in the comfort of my home since there is no need to travel to the hospital to see a doctor for consultation. In particular, videoconferencing is vital because it allows patients and doctors to interact remotely instead of seeking face-to-face appointments. This benefit becomes crucial in healthcare delivery, especially when there is a health crisis such as the COVID-19 pandemic that required social distancing as a way of controlling the spread.' [P.1].

3.2 Enhanced frequency and quality of communication. Another view that almost every interviewee in this study shared is that incorporating AI and telemedicine into healthcare systems enhances the frequency and quality of communication between patients and healthcare professionals. They noted that these technologies can provide the means by which healthcare professionals can tailor the messages to suit the patient's needs and preferences. Such messages were hailed as crucial to the patient's ability to adhere to professionals' advice and follow prescribed medication plans. The ability of these technologies to keep and validate patients' history was also hailed by most participants, who argued that such a history provides the foundation for patients and doctors to discuss what needs to be done and update the records in real time. As one of the patients argued:

'Having a discussion with a physician who has an updated history about you is encouraging because it shows that somebody cares about you and your condition and is ready to help you the best they can. This history also helps healthcare professionals tailor their messages to suit each patient and facilitate an engaging conversation. As such, it makes patients more proactive and an active part of the treatment processes, especially in adhering to medications and the doctor's instructions. I count myself as one of the patients who have become increasingly proactive about the management of my condition through the constructive engagements I have been having with my doctors using these technologies.' [P.3].

3.3 Improved patient-doctor relationship. Most healthcare professionals agreed that the use of AI and telemedicine in clinical settings has the potential to revolutionize how patients interact with physicians, thus boosting patient-doctor relationships. In particular, AI was cited as a crucial tool in this endeavor due to its potential to provide an automated mechanism for patients to have constructive engagements with healthcare providers. AI algorithms can help computers interpret patients' language and process it in a manner that facilitates effective communication. They can also help to analyze huge chunks of information and present it in a way that helps physicians make informed decisions concerning the best course of action to improve health outcomes and boost patients' satisfaction and positive experiences. According to most interviewees, constructive engagements between patients and healthcare providers are critical in fulfilling patients' needs and boosting their satisfaction levels. One of the doctors explained the following:

'I believe that AI and telemedicine hold the greatest potential to improve the patient-doctor relationship by facilitating constructive engagements between the patient and the doctor. Such engagements are vital to patients' satisfaction with healthcare services because they help doctors understand patients' needs and the most effective approach to addressing them. In the few years I have interacted with these technologies, I have learned that patients appreciate when the doctor shows she understands their needs and that everything is being done to address them.' [P.2].

3.4 Proactivity in care delivery. It emerged that using digital health technologies, such as AI and telemedicine, is critical in making healthcare delivery proactive instead of reactive. According to most of the healthcare professionals interviewed in this study, recent advancements in AI have necessitated the development of data analytic tools capable of helping healthcare providers devise a plan for staying in regular contact with their patients and ensuring that they are

adhering to the recommended medical attention. They also provide a mechanism for identifying patients' needs correctly and developing a strategy for addressing them based on their urgencies. In this case, the most severe cases are prioritized based on risk profile. As one of the doctors asserted:

'The widespread use of AI and telemedicine in clinical settings promises to make healthcare delivery more proactive by allowing physicians to devise effective mechanisms of identifying patients' needs and addressing them promptly based on severity or risk scores. These technologies also provide a means for monitoring the patients and ensuring that they are adhering to the prescribed medication to avoid complications. As such, cases of negligence are addressed before posing a danger to the patient's health. They also give physicians a comprehensive view of every patient, including their medical history, present condition, and the recommended course of action based on that information.' [P.13].

3.5 Quick discharge of patients. The administrators interviewed in this study cited the use of digital health technologies, such as AI and telemedicine, as one of the measures that can help boost the healthcare system's efficiency by streamlining the process of discharging patients from hospitals. They noted that the traditional ways of manually discharging patients were tedious because they relied heavily on paper trails and informal communication that contributed to disorganization. However, automation has allowed them to streamline the process and make it seamless. According to one of the administrators, *'automation using technologies such as AI is the best option that the healthcare system has of improving efficiency not only in the patient discharge process but also in all departments. I would recommend that the few technical and ethical issues that have prevented widespread adoption of AI in the healthcare sector be addressed to make care delivery more efficient.'* [P.10].

Apart from the administrators, patients also hailed these digital health technologies for improving the efficiency with which they have received services in different facilities that have automated some of their services. They agreed with the administrators that the efficiency of discharging patients has improved significantly through automation. One of them shared the following:

'I have had the benefit of experiencing the improved efficiency brought about by using digital healthcare technologies. Initially, admission and discharge were tedious and time-consuming because of the numerous manual processes involved. However, I recently took my father to the same facility, and I can confirm that things have changed and that it takes less time to accomplish all these things.' [P.5].

3.6 Ethical and privacy concerns. While all the participants agreed that AI and telemedicine have the potential to revolutionize healthcare delivery and make it more efficient, a significant number of them expressed concerns that the technologies are relatively new and there are several ethical and privacy concerns they must address to increase their chances of fulfilling this potential. In particular, the issue of data privacy and security was raised by most of the interviewees, who noted that the fear of data breaches and related malpractices is one of the primary reservations they have concerning the use of AI and telemedicine in clinical settings. To this end, addressing these concerns was considered necessary to increase patient satisfaction and experiences. One of the interviewees noted the following:

'The use of AI and telemedicine in healthcare delivery is inevitable in the modern era, where every sector is heading towards automation. However, these technologies are associated with significant ethical and privacy concerns that must be addressed for them to fulfill their

potential. Patients need to feel that their data privacy and security are safeguarded for them to trust these technologies and support their adoption and integration in healthcare systems.' [P.4].

Discussion

The principal aim of this study was to explore the contextual factors influencing the acceptance, implementation, and effectiveness of AI and telemedicine from the perspectives and experiences of patients and healthcare professionals. It sought to achieve three primary objectives: (1) explore healthcare professionals' perceptions and experiences with AI and telemedicine; (2) identify barriers and facilitators affecting the adoption and effective use of AI and telemedicine in clinical settings; and (3) understand patient experiences and satisfaction levels with AI and telemedicine interventions.

The healthcare professionals interviewed in the study had mixed feelings concerning AI and telemedicine because they highlighted both the benefits and drawbacks of using these digital health technologies. Among the identified benefits included enhancing patient participation and facilitating patient-centric care, cost and time saving, controlling the spread of infectious diseases, and boosting primary care and chronic illnesses management. It emerged that these technologies allow healthcare professionals to engage patients in their treatment and rehabilitation processes. In other words, they facilitate remote treatment, whereby the doctor attends to patients from the comfort of their homes without any of them having to travel. They also provide a mechanism for assigning patients responsibilities and allowing them to set goals concerning their health. This finding is consistent with many other studies that have investigated the perceptions of patients and healthcare professionals concerning the use of digital health technologies in clinical settings. For instance, a study conducted by Boulos et al. [10] established that these technologies have revolutionized the doctor-patient relationship by creating opportunities for patients to engage and

acquire more information that allows them to take part in the treatment process. However, some studies have found these technologies to be detrimental to the doctor-patient relationship by decreasing personal contact or face-to-face interactions between patients and healthcare professionals [11, 12]. These contradicting results underscore the need for further studies on the influence of digital health technologies on the doctor-patient relationship.

Cost and time saving were another benefit the participants associated with AI and telemedicine. It emerged that when the two technologies are applied in healthcare settings, patients and healthcare providers save both their time and cost because of improved efficiency and elimination of role duplications. This finding implies that using AI and telemedicine makes the healthcare system more efficient by improving its workers' productivity and saving the patients hassles associated with traveling from home to hospital. Past studies have also shown that cost and time saving are some of the primary benefits healthcare professionals associate with introducing new digital health technologies. For instance, Lugo et al. [13] examined the cost-effectiveness of managing obstructive sleep apnea using telemedicine and established that this technology made the process more cost-effective than routine hospital visits. Similarly, Eze et al. [14] investigated the cost and clinical effectiveness of using telemedicine and found that despite the set-up costs being a barrier to implementing this technology, it is more effective in the long run than face-to-face interactions.

The participants' views regarding these technologies also suggested that there were areas or healthcare departments where these technologies were more suited than others. It emerged that incorporating AI and telemedicine into healthcare facilities can revolutionize the healthcare system by helping control infectious diseases and boosting primary care and chronic illnesses management. Their suitability for these roles was highlighted during the COVID-19 outbreak

when restrictions of movement and physical distancing requirements meant that providing some services remotely was the most viable option for most healthcare providers. Research shows that the demand for primary health care (PHC) has risen considerably due to the increased number of people with chronic illnesses [15]. This increase has resulted in hospital overload, making remote services necessary for healthcare facilities. Peyroteo et al. [15] observed that using technologies such as AI and telemedicine would allow healthcare providers to monitor and track symptoms remotely without exposing the patients or themselves to infectious diseases. In particular, patients with chronic illness can have their conditions monitored in the comfort of their homes.

Despite the many benefits associated with AI and telemedicine, this study has shown that the technologies have several drawbacks based on the healthcare professionals' concerns. They expressed concerns such as misinformation and misdf iagnosis, unsuitability for emergency care, safety, and financial constraints. This finding indicates that AI and telemedicine can only achieve their maximum potential by addressing these concerns. Other barriers they must overcome include concerns about voluminous data and strict regulations, skepticism/fears about data protection, infrastructural and technical shortcomings, resistance to change, and skills gaps. The identification of voluminous data as a barrier to the adoption of AI and telemedicine was a surprising finding because it is expected that technologies such as AI and telemedicine improve users' ability to analyze voluminous data and derive meaning from it. Some doctors expressed concerns that these technologies could lead to increased workflows and the amount of data they must deal with in their day-to-day operations. These concerns indicate the possibility of a lack of awareness among the target users in the healthcare sector.

Most of the barriers identified in this study can be addressed through various mechanisms that the participants identified as the primary facilitators for adopting and effectively using AI and

telemedicine. They include creating awareness about AI and telemedicine, providing thorough training and education to healthcare professionals, engaging healthcare providers in developing and implementing digital health technologies, and incentivizing digital health technologies. For instance, barriers related to fears about data protection, resistance to change, misinformation and misdiagnosis, and skills gaps can be addressed through increased awareness about AI and telemedicine, thorough training and education to healthcare professionals, engaging stakeholders in the development and implementation processes, and incentivizing the use of these technologies. If healthcare facilities can minimize the barriers and maximize the facilitators, the positive impacts patients associate with AI and medicine (convenience, enhanced frequency and quality of communication, improved patient-doctor relationships, and proactivity in care delivery) can be realized. In support of this view, Borges do Nascimento et al. [16] established that the adoption and effectiveness of digital health technologies can be enhanced by maximizing the facilitators, including tailoring training and awareness programs to the needs of the target users (healthcare professionals).

Strengths and Limitations

This study's primary strength is being the first to comprehensively investigate the contextual factors influencing the adoption and effective use of digital health technologies like AI and telemedicine in clinical settings. Its use of the qualitative approach allowed us to get to the depth of the research problem, whereby a comprehensive understanding of the dynamics involved in the acceptance, implementation, and effectiveness of AI and telemedicine were exposed. Understanding these factors from the perspectives of patients and healthcare professionals is essential because their views can provide the foundation from which the adoption and effectiveness of these technologies can be enhanced. They can also facilitate policy development by expanding

the policymakers' knowledge of the barriers and facilitators influencing the adoption and effective use of AI and telemedicine.

Despite these strengths, the study was not free of downsides. First, the sample size used was not sufficient to allow generalization of the findings. It is worth noting that only 15 individuals participated in the interviews. These individuals were derived from hospitals in the two UAE emirates (Abu Dhabi and Dubai), suggesting that the scope was limited to the UAE setting. As such, the results could not be generalized beyond the UAE or other settings outside the two Emirates. The other limitation concerned the use of purposive sampling to select the participants. This technique involves using predetermined criteria or qualifications to choose the study subjects. In this case, the participants had to be individuals with direct experience in AI and telemedicine or have interacted with the two technologies in the course of providing or receiving healthcare services. This limitation suggests that the views of individuals who have not interacted with AI and telemedicine directly were not captured. Healthcare professionals working in institutions that have not implemented either of these technologies were also excluded, thus limiting the results' generalizability.

Future research can respond to these limitations and findings in various ways. First, they can enhance the findings' generalizability by increasing the sample size and expanding the scope beyond the two Emirates (Abu Dhabi and Dubai) and outside the UAE to see if similar findings can be made. Future studies can also incorporate other sampling techniques to ensure that the research population is as diverse as possible. This approach would also ensure that diverse views can be obtained even from people who have not interacted with these technologies directly but have an opinion regarding their adoption in healthcare settings. Furthermore, since healthcare professionals working in institutions that have implemented AI or telemedicine were not included

in this study, future researchers should include them in their studies to understand why these institutions have not embraced these technologies.

Conclusions

The principal aim of this study was to explore the contextual factors influencing the acceptance, implementation, and effectiveness of AI and telemedicine in clinical settings. Based on the responses from healthcare professionals, patients, hospital administrators, and IT specialists who have interacted with the two technologies, it emerged that AI and telemedicine are attributed to several benefits. They include cost and time saving, convenience, enhancing patient participation and facilitating patient-centric care, controlling the spread of infectious diseases, boosting primary care and managing chronic illnesses, and improving doctor-patient relationships. Despite these benefits, the adoption and effective use of these technologies are hampered by several barriers/challenges, including misinformation and misdiagnosis, safety concerns, unsuitability for emergency care, infrastructural and technical shortcomings, resistance to change, skills gap, and financial constraints.

Some of these barriers can be addressed using the facilitators identified by the participants, including creating awareness about AI and telemedicine, providing thorough training to healthcare professionals, engaging stakeholders in the design and implementation processes, and incentivizing digital health technologies. Investing in the infrastructure required to implement these technologies can also help to boost not only their adoption but also their effective use in clinical settings. Furthermore, the ethical and privacy concerns highlighted by the participants suggest the need to strengthen the legal framework governing these technologies' use to enhance the target users' confidence and facilitate their widespread adoption. These findings have significant theoretical and practical implications. Apart from expanding the available literature on

the factors influencing the adoption and effective use of digital health technologies such as AI and telemedicine, they provide evidence-based insights that healthcare institutions can use to improve the effectiveness of AI and telemedicine. The study also sheds light on what institutions that have not implemented these technologies can do to stimulate adoption and ensure that healthcare professionals are prepared and motivated to use digital health technologies.

Acknowledgments

The author wishes to thank all the participants (healthcare professionals and patients) who agreed to participate in this study. Their time and invaluable contributions during the data collection process are highly appreciated.

References

1. Győrffy Z, Radó N, Mesko B. Digitally engaged physicians about the digital health transition. PLoS One. 2020 Sep 28; 15(9):e0238658. doi: 10.1371/journal.pone.0238658.

2. Wernhart A, Gahbauer S, Haluza D. eHealth and telemedicine: Practices and beliefs among healthcare professionals and medical students at a medical university. PloS one. 2019 Feb 28; 14(2):e0213067. doi: 10.1371/journal.pone.0213067.

3. Shahid N, Rappon T, Berta W. Applications of artificial neural networks in health care organizational decision-making: A scoping review. PloS one. 2019 Feb 19; 14(2):e0212356. doi: 10.1371/journal.pone.0212356.

4. Phuong J, Ordóñez P, Cao J, Moukheiber M, Moukheiber L, Caspi A, Swenor BK, Naawu DK, Mankoff J. Telehealth and digital health innovations: A mixed landscape of access. PLOS Digital Health. 2023 Dec 15; 2(12):e0000401. doi: 10.1371/journal.pdig.0000401.

5. Doyle L, McCabe C, Keogh B, Brady A, McCann M. An overview of the qualitative descriptive design within nursing research. Journal of research in nursing. 2020 Aug; 25(5):443-55. doi: 10.1177/1744987119880234.

6. Peterson JS. Presenting a qualitative study: A reviewer's perspective. Gifted Child Quarterly. 2019 Jul; 63(3):147-58. doi: 10.1177/0016986219844789.

7. Saunders B, Sim J, Kingstone T, Baker S, Waterfield J, Bartlam B, Burroughs H, Jinks C. Saturation in qualitative research: exploring its conceptualization and operationalization. Quality & quantity. 2018 Jul; 52:1893-907. doi: 10.1007/s11135-017-0574-8.

8. Braun V, Clarke V. Using thematic analysis in psychology. Qualitative research in psychology. 2006 Jan 1; 3(2):77-101. doi: 10.1191/1478088706qp063oa.

9. López-Zerón G, Bilbao-Nieva MI, Clements KA. Conducting member checks with multilingual research participants from diverse backgrounds. Journal of Participatory Research Methods. 2021 Jul 21; 2(2). doi: 10.35844/001c.24412.

10. Boulos MN, Wheeler S, Tavares C, Jones R. How smartphones are changing the face of mobile and participatory healthcare: An overview, with example from eCAALYX. Biomedical engineering online. 2011 Dec; 10:1-4. doi: 10.1186/1475-925X-10-24.

11. Graefen B. The doctor-patient relationship in the era of e-health. ESTÜDAM Halk Sağlığı Dergisi. 2023 May 5;8(2):220-9. doi: 10.35232/estudamhsd.1239685.

12. Botrugno C. Information technologies in healthcare: Enhancing or dehumanising doctor–patient interaction?. Health. 2021 Jul;25(4):475-93. doi: 10.1177/136345931989.

13. Lugo VM, Garmendia O, Suarez-Girón M, Torres M, Vázquez-Polo FJ, Negrín MA, Moraleda A, Roman M, Puig M, Ruiz C, Egea C. Comprehensive management of obstructive sleep apnea by telemedicine: clinical improvement and cost-effectiveness of a virtual sleep unit. A randomized controlled trial. PLoS One. 2019 Oct 24;14(10):e0224069. doi: 10.1371/journal.pone.0224069.

14. Eze ND, Mateus C, Cravo Oliveira Hashiguchi T. Telemedicine in the OECD: an umbrella review of clinical and cost-effectiveness, patient experience and implementation. PloS one. 2020 Aug 13;15(8):e0237585. doi: 10.1371/journal.pone.0237585.

15. Peyroteo M, Ferreira IA, Elvas LB, Ferreira JC, Lapão LV. Remote monitoring systems for patients with chronic diseases in primary health care: systematic review. JMIR mHealth and uHealth. 2021 Dec 21;9(12):e28285. doi: 10.2196/28285. PMID: 34932000.

16. Borges do Nascimento IJ, Abdulazeem H, Vasanthan LT, Martinez EZ, Zucoloto ML, Østengaard L, Azzopardi-Muscat N, Zapata T, Novillo-Ortiz D. Barriers and facilitators to

utilizing digital health technologies by healthcare professionals. NPJ digital medicine. 2023 Sep 18;6(1):161. doi: 10.1038/s41746-023-00899-4.
