# Supplementary material for: Navigating digital frontiers in UAE healthcare: A qualitative exploration of healthcare professionals’ and patients’ experiences with AI and telemedicine
Source: PLOS Digit Health. 2025 Apr 8;4(4):e0000586. doi: 10.1371/journal.pdig.0000586 (PMC11991283; doi:10.1371/journal.pdig.0000586)
Supplement: Attachment — Submitted filename: Article Review-PDIG-D-24-00287.pdf [file pdig.0000586.s003.pdf]

Navigating Digital Frontiers in Healthcare: A Qualitative Exploration of Healthcare Professionals' and Patients' Experiences with AI and Telemedicine

A. SUMMARY OF THE RESEARCH FINDINGS AND OVERALL IMPRESSION

i. Summary of the Research findings

Abstract section

The abstract effectively summarizes the study, providing a clear outline of the research objectives, methodology, and key findings. It highlights the benefits of AI and telemedicine, as well as the barriers and facilitators affecting their adoption. However, the abstract could be improved by including specific details about the study's location and the unique contribution of the research to existing literature, which would provide readers with a clearer context. Future work and the policy implications from the findings are also missing and need to be included

Other Sections

The introduction section has provided a comprehensive background on digital health technologies and their impact on healthcare. It sets the stage well by discussing AI and telemedicine's potential and the paradigm shift they represent in healthcare delivery. However, the section could be improved by specifying the research gap more explicitly and clearly stating how this study addresses it.

Looking at the methods section, it is well-detailed, describing the qualitative descriptive research design, sampling procedure, and data collection methods. The use of semi-structured interviews and thematic analysis is appropriate for the study's objectives. However, the study's limitations in sampling, such as the small sample size and focus on only two Emirates in the UAE, could be highlighted earlier in this section. Including a justification for choosing a qualitative approach over a mixed-methods approach could also strengthen the methodological rigor.

The results section has provided a thorough presentation of the findings, organized around the study's key objectives. Themes are well-defined, and quotes from participants effectively illustrate the insights gathered. The section successfully captures the complexities of healthcare professionals' and patients' experiences with AI and telemedicine. However, some of the findings, particularly those related to specific barriers, could be more critically examined to explore deeper implications.

The discussion section interprets the results well, linking them to existing literature and highlighting the practical implications of the findings. It effectively addresses the benefits and drawbacks of AI and telemedicine and provides insights into the barriers and facilitators of their adoption. However, the discussion could be enriched by exploring the findings' theoretical implications more deeply and comparing the study results with those from different contexts outside the UAE.

The conclusion concisely summarizes the main findings and offers practical recommendations to improve the adoption and effectiveness of AI and telemedicine. However, it would be beneficial to suggest specific actions or policy changes needed to address the identified barriers, such as targeted training programs or infrastructural investments.

Overall Impression

The manuscript provides valuable insights into the contextual factors influencing the adoption of AI and telemedicine in clinical settings. It makes a significant contribution by exploring the perspectives of both

healthcare professionals and patients. The thematic approach used in the analysis is appropriate and allows for a nuanced understanding of the challenges and opportunities associated with digital health technologies.

B. SPECIFIC AREAS OF IMPROVEMENT

- ✓ Add missing information in the abstract
- ✓ There is need for more clarity in stating the research gap in the introduction section. The authors should more explicitly identify the research gap and clearly articulate how this study fills that gap compared to existing studies.
- ✓ Readers would like to see some theoretical implications of the findings, particularly in relation to existing models of technology adoption in healthcare.
- ✓ The conclusion should provide more specific policy recommendations based on the findings, such as initiatives to improve training, address infrastructural deficits, or develop legal frameworks to protect data privacy.
