# Supplementary material for: Navigating digital frontiers in UAE healthcare: A qualitative exploration of healthcare professionals’ and patients’ experiences with AI and telemedicine
Source: PLOS Digit Health. 2025 Apr 8;4(4):e0000586. doi: 10.1371/journal.pdig.0000586 (PMC11991283; doi:10.1371/journal.pdig.0000586)
Supplement: Attachment — Submitted filename: Rebuttal Letter.docx [file pdig.0000586.s004.docx]

**Rebuttal Letter**
18 November 2024

Shannon Freeman, PhD
Academic Editor
PLOS Digital Health

**Re: Manuscript Title: Navigating digital frontiers in UAE healthcare: A qualitative exploration of healthcare professionals' and patients' experiences with AI and telemedicine**
**Manuscript ID: PDIG-D-24-00287**

Dear Dr. Freeman,

We would like to extend our sincere thanks to you and the reviewers for the insightful feedback provided on our manuscript titled *"Navigating digital frontiers in UAE healthcare: A qualitative exploration of healthcare professionals' and patients' experiences with AI and telemedicine."* We have carefully considered each of the reviewers' comments and have undertaken substantial revisions to address all concerns. Below, we provide a point-by-point response to each comment, outlining the changes made and the rationale behind our approach. All changes have been highlighted in green in the revised manuscript for ease of reference, as we felt using track changes would make the document cumbersome and less clear due to extensive restructuring, particularly in the results and discussion chapters.

**Reviewer #1 Comments and Responses**

1. **Comment:** The manuscript needs to be adapted to the journal's standards, including formatting, line numbers, font sizes, etc.
   *Response:* The manuscript has been reformatted according to PLOS Digital Health's submission guidelines, ensuring appropriate font sizes, line numbering, and alignment with the journal’s style for headings and overall layout.
2. **Comment:** Additional comments were provided directly within the manuscript for clarity and improvements.

*Response:* We have carefully addressed each comment made directly in the manuscript by Reviewer #1. All suggested edits, clarifications, and recommendations have been reviewed and integrated where appropriate. This includes ensuring consistent terminology, enhancing clarity in specific sections, and refining the presentation of key findings. We appreciate the detailed feedback and believe it has significantly strengthened the quality of the manuscript.

**Reviewer #2 Comments and Responses**

1. **Comment:** The abstract needs subheadings for clarity.
   *Response:* Subheadings have been added to the abstract to clearly demarcate the sections, including Objectives, Methods, Results, and Conclusions. This change improves readability and aligns with the journal's recommendations for structuring abstracts.
2. **Comment:** The title and abstract should explicitly reflect the UAE context of the study.
   *Response:* The title and abstract have been revised to emphasize the study's focus on the UAE healthcare system and the unique cultural and contextual factors explored in the research.
3. **Comment:** Clarification is needed on qualitative research terminology, focusing on perceptions rather than effectiveness.
   *Response:* Throughout the manuscript, references to "effectiveness" have been modified to reflect "perceptions" and "attitudes" as appropriate for qualitative research. This change better represents the scope and nature of the study.
4. **Comment:** Separation of AI and telemedicine concepts is necessary.
   *Response:* The results and discussion sections have been restructured to differentiate between AI and telemedicine, providing a clearer analysis of their distinct roles, benefits, and challenges. This separation ensures a more comprehensive interpretation and aligns with the feedback provided.
5. **Comment:** More detailed synthesis in the introduction is required.
   *Response:* The introduction has been expanded to provide a comprehensive overview of relevant literature, a clear statement of the research gap, and the significance of this study within the UAE healthcare context.
6. **Comment:** Justification for the sample size and data saturation is needed.
   *Response:* The methodology section has been revised to include a detailed explanation of how data saturation was achieved with our sample size and why this sample was deemed sufficient to explore the study’s objectives.
7. **Comment:** Age distribution and potential biases in participant responses should be addressed.
   *Response:* The discussion has been updated to acknowledge and elaborate on the influence of participant age distribution on the study's findings, providing a balanced view of potential biases.
8. **Comment:** Theoretical framework and contextual barriers/facilitators need clearer discussion.
   *Response:* We have clarified the theoretical basis of the study and explained how identified barriers and facilitators align with implementation science frameworks, offering further depth to the discussion.
9. **Comment:** Address subjectivity in qualitative data analysis.
   *Response:* Additional information has been provided on strategies used to minimize subjectivity, including researcher reflexivity, peer review of codes, and the lead researcher’s extensive experience with qualitative methodologies.
10. **Comment:** Include ethical approval details and reference number.
    *Response:* Ethical approval details have been added, specifying approval from the Ministry of Health and Prevention (MOHAP) Ethical Committee with reference number MOHAP/REC/2024/00123.
11. **Comment:** The results section has a description of questions asked to participants, which will be better suited in the methods section than in the results.

*Response:* We agree with this observation. The interview questions described in the results section have been relocated to the methodology section to ensure clarity and improve the focus on presenting findings in the results chapter.

12. **Comment:** I am concerned that authors may overinterpret some of the data analysis because they were making a claim that might not be fully grounded in the qualitative data. For example, the results section should always refer to participants’ perceptions “Participants thought/saw/believed/suggested/agreed/felt etc” rather than making a substantial claim about AI (e.g., “It [telemedicine] also allows them to give more intensive care to the patients who require such care because their needs can be assessed more comprehensively.”)

*Response:* We have revised the results section to ensure that claims are directly tied to participants' perceptions and experiences. Throughout the manuscript, we have reframed statements to reflect participants' viewpoints, using phrasing such as “Participants perceived,” “Participants suggested,” and “Participants felt” to maintain alignment with qualitative reporting standards. This adjustment ensures that interpretations are grounded in the qualitative data presented.

13. **Comment:** The presentation of the quotes is also without the authors’ interpretation. It is almost as if the quotes are inserted for the readers’ interpretation. Instead, a careful description of the data analysis outcomes should be outlined with quotes to demonstrate the interpretations. Some quotes even formulate a stand-alone paragraph, which is unacceptable in qualitative research write-ups.

*Response:* We have revised the presentation of quotes in the results section. Each quote is now accompanied by a detailed interpretation, providing context and linking it to the themes being discussed. Stand-alone quotes have been integrated into the narrative, and explanations now clarify how the quotes support the thematic findings.

14. **Comment:** Reading through the analysis, it also does not separate AI from telemedicine, and while these two technologies are very different in terms of their applications, it isn't easy to understand what participants were referring to. The analysis is lacking sufficient depth in that respect.

*Response:* To address this concern, we have reorganized and clarified sections of the results and discussion to distinctly separate the findings related to AI from those concerning telemedicine. This distinction adds depth to the analysis and allows for a clearer understanding of how participants perceive each technology.

15. **Comment:** There is an unnecessary level of repetitiveness throughout the manuscript. For example, there is no need to repeat the study aims at the beginning of the discussion. Instead, the authors should reiterate the main findings and explain them.

*Response:* We have removed the repetition of the study aims at the beginning of the discussion section. Instead, the main findings are reiterated and expanded upon to provide a seamless transition into the detailed interpretation and implications of the results. We believe this change enhances the clarity and flow of the discussion.

16. **Comment:** As many of the themes reflect ideas previously identified in other studies, such as cost or convenience, the discussion section would benefit from the outline of the new or novel knowledge captured in this study that had not been previously uncovered. I think that would make this study unique and citable. Thus, more attention should be dedicated to the unique and odd findings in the study rather than repeating what was already reported in other qualitative studies and systematic reviews.

*Response:* We have revised the discussion section to place greater emphasis on the novel findings unique to this study, particularly those reflecting UAE-specific challenges, cultural influences, and context-specific barriers and facilitators to digital health technology adoption. This enhancement highlights the study's contribution to existing literature and underscores the distinctive insights provided by this research.

17. **Comment:** In the discussion – strengths and weaknesses the authors claim “This study’s primary strength is being the first to comprehensively investigate the contextual factors influencing the adoption and effective use of digital health technologies like AI and telemedicine in clinical settings.” However, this is not the first qualitative study to investigate this. [Young, A. T., Amara, D., Bhattacharya, A., & Wei, M. L. (2021). Patient and general public attitudes towards clinical artificial intelligence: a mixed methods systematic review. The lancet digital health, 3(9), e599-e611.] The authors may want to review the existing literature on the subject to reassess their novelty.

*Response:* We appreciate this feedback. The statement regarding the novelty of our study has been revised to clarify that while other studies have explored related themes, our study provides a comprehensive focus on the UAE context, offering insights that are not widely represented in existing literature. We have also reviewed and cited relevant studies, including the work by Young et al. (2021), to provide a balanced discussion of existing contributions to this area of research.

18. **Comment:** I agree with the authors about the study limitations and the narrow focus on the UAE. I would suggest rewriting the report to focus on sufficiently covering the UAE context in the introduction, title, and abstract so that researchers can quickly identify its scope. I would also recommend adjusting the discussion section to fit it within the UAE context so that others can understand how the geo-political issues influence those perceptions. I think a major rewrite is therefore needed so that this article is carefully placed within its healthcare context.

*Response:* We have thoroughly revised the introduction, title, and abstract to ensure that the UAE context is clearly highlighted. Additionally, the discussion section has been adjusted to incorporate a more in-depth examination of how UAE-specific geopolitical and cultural factors influence perceptions and the adoption of digital health technologies. This ensures that the study’s scope and contributions are immediately identifiable and relevant to readers.

19. **Comment:** There is also a need for a stronger ‘implications’ subsection from this study outlining how this knowledge can be applied in clinical settings.

*Response:* A dedicated 'Implications for Practice and Policy' subsection has been added to the discussion to address this comment. This section outlines specific recommendations for applying the study’s findings in clinical settings, including training programs, awareness initiatives, and policy reforms, to enhance the adoption and effective use of digital health technologies in the UAE.

**Reviewer #3 Comments and Responses**

 **Abstract**
**Comment:** The abstract effectively summarizes the study, providing a clear outline of the research objectives, methodology, and key findings. However, it could be improved by including specific details about the study's location and the unique contribution of the research to existing literature, which would provide readers with a clearer context. Future work and policy implications from the findings are also missing and need to be included.
*Response:* The abstract has been refined to include specific details about the UAE context and highlight the study's unique contributions to the literature. Additionally, policy implications and areas for future research have been succinctly outlined to offer a clearer and more comprehensive overview of the study's impact.

 **Introduction**
**Comment:** The introduction section provides a comprehensive background on digital health technologies and their impact on healthcare. However, the section could be improved by specifying the research gap more explicitly and clearly stating how this study addresses it.
*Response:* The introduction has been revised to clarify the research gap and explicitly articulate how this study addresses it, distinguishing it from existing research and highlighting its unique focus on contextual factors within the UAE.

 **Methods**
**Comment:** Looking at the methods section, it is well-detailed, describing the qualitative descriptive research design, sampling procedure, and data collection methods. However, the study's limitations in sampling, such as the small sample size and focus on only two Emirates in the UAE, could be highlighted earlier in this section. Including a justification for choosing a qualitative approach over a mixed-methods approach could also strengthen the methodological rigor.
*Response:* The methods section now includes a discussion of the study’s sampling limitations, highlighting the focus on two Emirates and the relatively small sample size. Additionally, we have included a justification for employing a qualitative descriptive approach, emphasizing its appropriateness for capturing in-depth perspectives from healthcare professionals and patients.

** Results**
**Comment:** The results section has provided a thorough presentation of the findings, organized around the study's key objectives. Themes are well-defined, and quotes from participants effectively illustrate the insights gathered. However, some of the findings, particularly those related to specific barriers, could be more critically examined to explore deeper implications.
*Response:* The results section has been restructured to provide a clearer separation of AI and telemedicine themes, as recommended. The findings related to specific barriers have been critically examined, with additional depth added to explore their implications.

 **Discussion**
**Comment:** The discussion interprets the results well, linking them to existing literature and highlighting the practical implications of the findings. However, the discussion could be enriched by exploring the findings' theoretical implications more deeply and comparing the study results with those from different contexts outside the UAE.
*Response:* The discussion has been expanded to include a detailed exploration of theoretical implications and a comparison of the study findings with results from different geographical contexts. This broader perspective enriches the discussion and situates our findings within the global discourse on digital health technologies.

 **Conclusion**
**Comment:** The conclusion concisely summarizes the main findings and offers practical recommendations to improve the adoption and effectiveness of AI and telemedicine. However, it would be beneficial to suggest specific actions or policy changes needed to address the identified barriers, such as targeted training programs or infrastructural investments.
*Response:* The conclusion has been revised to provide specific policy recommendations based on the findings. These include targeted training programs, awareness initiatives, infrastructural improvements, and strategies to address data privacy concerns.

We hope that the revisions made have addressed all comments to your satisfaction. We are grateful for the opportunity to improve our manuscript and believe it is now much stronger as a result of the reviewers' valuable feedback.

Thank you for your time and consideration. We look forward to your response.

Sincerely,
**Azza Alkaabi**
[aalkaabi@ra.ac.ae](mailto:aalkaabi@ra.ac.ae)
ORCID ID: 0009-0000-3827-1808

**Deena Elsori**
[delsori@ra.ac.ae](mailto:delsori@ra.ac.ae)
ORCID ID: 0000-0002-1063-5101
